# Clinical-grade N-(4-[^18^F]fluorobenzoyl)-interleukin-2 for PET imaging of activated T-cells in humans

**DOI:** 10.1186/s41181-019-0062-7

**Published:** 2019-07-17

**Authors:** Elly L. van der Veen, Inês F. Antunes, Petra Maarsingh, Janet Hessels-Scheper, Rolf Zijlma, Hendrikus H. Boersma, Annelies Jorritsma-Smit, Geke A. P. Hospers, Elisabeth G. E. de Vries, Marjolijn N. Lub-de Hooge, Erik F. J. de Vries

**Affiliations:** 1 Department of Medical Oncology, University of Groningen, University Medical Center Groningen, Hanzeplein 1, P.O. Box 30.001, 9700 RB Groningen, The Netherlands; 2 Department of Nuclear Medicine and Molecular Imaging, University of Groningen, University Medical Center Groningen, Hanzeplein 1, P.O. Box 30.001, 9700 RB Groningen, The Netherlands; 3 Department of Clinical Pharmacy and Pharmacology, Hanzeplein 1, P.O. Box 30.001, 9700 RB Groningen, The Netherlands

**Keywords:** Positron emission tomography, Interleukin-2, Molecular imaging, T-cells, Inflammation, Immunotherapy, Fluor-18

## Abstract

**Background:**

Molecular imaging of immune cells might be a potential tool for response prediction, treatment evaluation and patient selection in inflammatory diseases as well as oncology. Targeting interleukin-2 (IL2) receptors on activated T-cells using positron emission tomography (PET) with N-(4-[^18^F]fluorobenzoyl)-interleukin-2 ([^18^F]FB-IL2) could be such a strategy. This paper describes the challenging translation of the partly manual labeling of [^18^F]FB-IL2 for preclinical studies into an automated procedure following Good Manufacturing Practices (GMP), resulting in a radiopharmaceutical suitable for clinical use.

**Methods:**

The preclinical synthesis of [^18^F]FB-IL2 was the starting point for translation to a clinical production method. To overcome several challenges, major adaptations in the production process were executed. The final analytical methods and production method were validated and documented. All data with regards to the quality and safety of the final drug product were documented in an investigational medicinal product dossier.

**Results:**

Restrictions in the [^18^F]FB-IL2 production were imposed by hardware configuration of the automated synthesis equipment and by use of disposable cassettes. Critical steps in the [^18^F]FB-IL2 production comprised the purification method, stability of recombinant human IL2 and the final formulation. With the GMP compliant production method, [^18^F]FB-IL2 could reliably be produced with consistent quality complying to all specifications.

**Conclusions:**

To enable the use of [^18^F]FB-IL2 in clinical studies, a fully automated GMP compliant production process was developed. [^18^F]FB-IL2 is now produced consistently for use in clinical studies.

**Electronic supplementary material:**

The online version of this article (10.1186/s41181-019-0062-7) contains supplementary material, which is available to authorized users.

## Background

Molecular imaging of immune cells for diagnosis and therapy evaluation in inflammatory and infectious diseases has been investigated for decades, but recently this field has expanded to oncology. The impressive anti-tumor effects of immunotherapeutics has resulted in a growing interest in immune cells and their role in tumor responses (Wykes & Lewin, 2018; van der Veen et al., 2018). Many immunotherapeutics are based on the activation of effector T-cells. Therefore, targeting these activated T-cells specifically with a radiolabeled imaging probe, might be a potential molecular imaging strategy in this context. The interleukin-2 (IL2) receptor, consisting of three subunits CD25, CD122 and CD132, is mainly expressed by these activated effector T-cells and by a subpopulation of regulatory T-cells (Malek & Castro, 2010). Molecular imaging of IL2 receptors using radiolabeled recombinant human IL2 could be a strategy to track activated T-cells expressing the IL2 receptor. In the past, IL2 receptors have been imaged using single photon emission computed tomography (SPECT) with technetium-99 m (^99m^Tc) or iodine-123 (^123^I) labeled IL2 analogues. Imaging could detect T-cell infiltration in patients with melanoma, carcinoma and various inflammatory disorders (Signore et al., 2004; Loose et al., 2008; Signore et al., 2003; D’Alessandria et al., 2010; Hubalewska-Dydejczyk et al., 2009). However, SPECT has a low spatial resolution and sensitivity, which makes it difficult to detect small lesions or lesions with low to moderate T-cell infiltration. Moreover, absolute quantification of the imaging signal is difficult with SPECT. Furthermore, for the purpose of early response prediction is it important to not only to detect T-cells, but also to be able to quantify the signal. To overcome the limitations of SPECT, the PET tracer N-(4-[^18^F]fluorobenzoyl)-interleukin-2 ([^18^F]FB-IL2) was developed. In vivo preclinical studies in mice showed that [^18^F]FB-IL2 is stable in plasma and [^18^F]FB-IL2 PET could detect CD25-positive human and murine T-cells as well as migration of these T-cells to distant sites of inflammation (Di Gialleonardo et al., 2012a). In immune competent rats, the accumulation of [^18^F]FB-IL2 correlated with the number of injected activated CD25-positive human T-cells (Di Gialleonardo et al., 2012b).

To bring this interesting PET tracer to the clinic, the production method needed to be adapted to suit strict regulations for production of radiopharmaceuticals. National guidelines in different EU countries impose to produce radiopharmaceuticals according to Good Manufacturing Practices (GMP) guidelines (Bormans et al., 2017). With these guidelines the quality of the radiopharmaceutical can be warranted, resulting in a radiopharmaceutical suitable for human use. Not only a robust and consistent production process is needed, but also final formulation must be of consistent quality and composition. Furthermore, production of the radiopharmaceutical suitable for human use implies the use of higher amounts of radioactivity and as a result protection of the operator from exposure to radiation is required. It requires that the partly manual production method developed and used in a research and development (R&D) setting is converted into a fully automated GMP compliant production method. Moreover, the final formulation needs to be safe for human use and additionally suitable purification and sterilization methods are required.

**Fig. 1:**
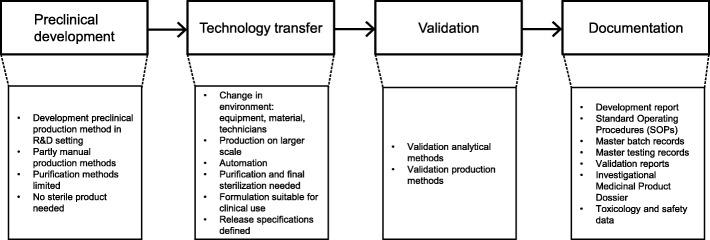
Development path and characteristics for radiopharmaceuticals

Guided by the phases of the development path of radiopharmaceuticals (Fig. 1) we here describe the challenges encountered during the translation to the GMP environment. Solutions for the encountered problems and the consequent changes in the production method are described. These modifications have resulted in a GMP compliant production process for the radiopharmaceutical suitable for first-in-human clinical use.

## Methods

### Preclinical development

**Fig. 2:**
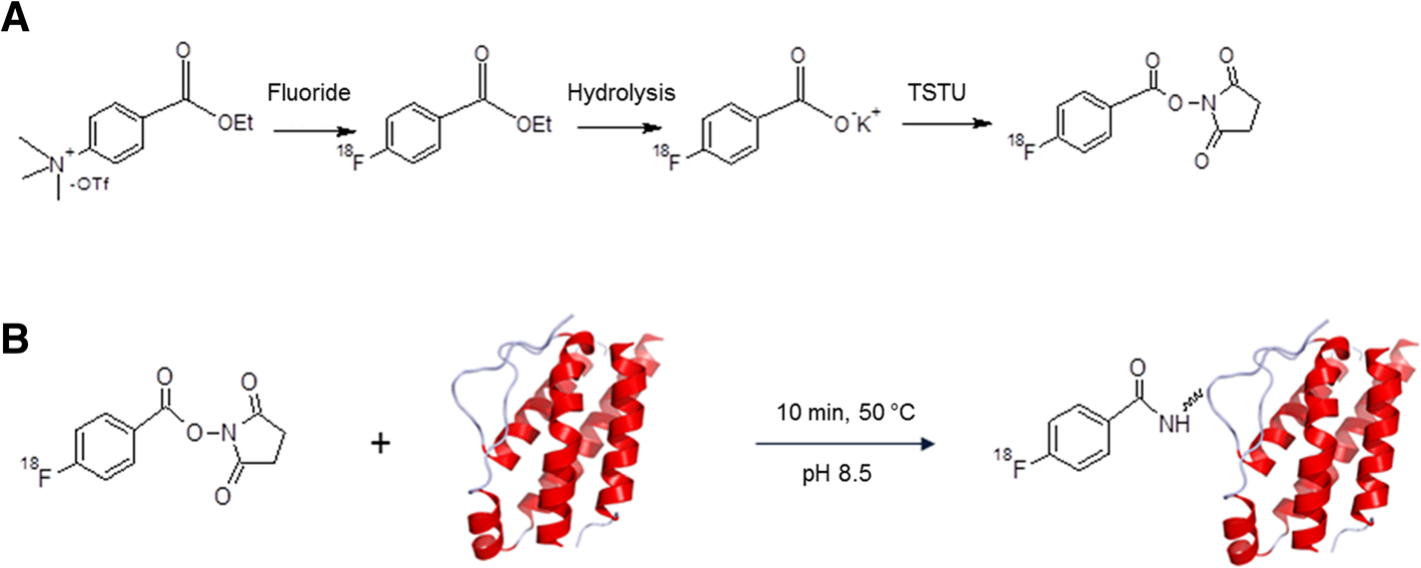
Overview of the synthesis steps of [^18^F]FB-IL2, consisting of 2 steps: **a**) Preparation of [^18^F]SFB and **b**) conjugation of [^18^F]SFB to IL2

The production method of [^18^F]FB-IL2 developed for preclinical studies has been described by Di Gialleonardo et al. (Di Gialleonardo et al., 2012b). In short, this production method consists of four steps as depicted in Fig. 2. First the precursor N-succinimidyl 4-[^18^F]fluorobenzoate ([^18^F]SFB) is produced in three steps: nucleophilic substitution of the ammonium group of ethyl 4-(trimethylammonium)benzoate triflate salt by a [^18^F]fluorine atom, hydrolysis of the ethyl ester and (Malek & Castro, 2010) formation of the succinimidyl ester. In this preclinical method the purification of [^18^F]SFB was performed using an Oasis HLB Sep-Pak cartridge.

In the fourth step, [^18^F]SFB is conjugated to recombinant human IL2. After the conjugation reaction, [^18^F]FB-IL2 was purified by high-performance liquid chromatography (HPLC) using an Elite LaChrom Hitachi L-7100 pump system with an Econosphere C18-column (10 μm, 250 mm × 10 mm) equipped with both ultraviolet (UV) detection (Elite LaChrom VWR L-2400 UV detector set at 254 nm; Hitachi) and a Bicron radioactivity monitor. Gradient elution was performed using a mixture of 0.1% aqueous trifluoroacetic acid and 0.1% trifluoroacetic acid (TFA) in ethanol. The product was collected from HPLC in approximately 55% ethanol in water. Thereafter, the product was diluted with 0.9% of saline in order to decrease the percentage of ethanol to lower than 10% for the subsequent pre-clinical studies.

### Technology transfer

#### [^18^F]SFB and [^18^F]FB-IL2 production

The development report with the described preclinical production method for [^18^F]FB-IL2 was the starting point for the translation to a production method for human use. Critical steps known from the preclinical development have been taken into account in the design of the clinical production method. A Modular-Lab PharmTracer Eckert & Ziegler synthesis module (4-fold and 6-fold cassette) was used for the GMP compliant design of the production method of [^18^F]SFB and [^18^F]FB-IL2. This module is equipped with two ovens and a single HPLC system, analogous to the previous described preclinical system.

**Fig. 3:**
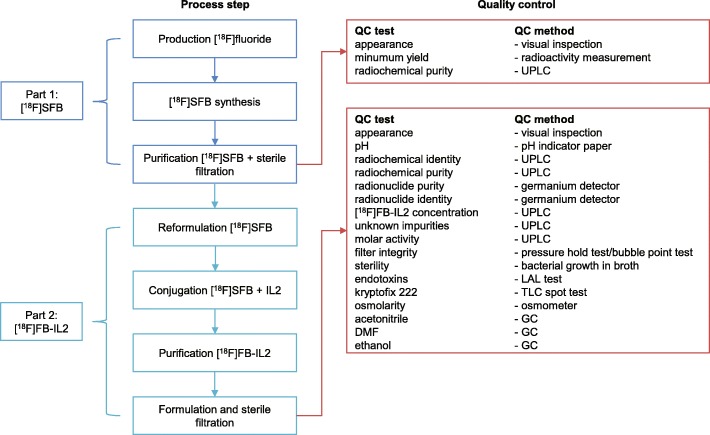
Flow chart GMP [^18^F]FB-IL2 manufacturing, including quality control requirements and methods. Abbreviations: UPLC: ultra-performance liquid chromatography; TLC: thin-layer chromatography; LAL: limulus amebocyte lysate; DMF: N,N-dimethylformamide; GC: gas chromatography

During implementation of the automated production method, hardware limitations were encountered, as will be described in the results section. Important was the change of [^18^F]SFB production to a non-classified hot cell and room with a Zymark robotic system. After purification by HPLC, sterilization by 0.2 μm filtration and quality control, [^18^F]SFB is transferred to a class C hot cell in a class C cleanroom. Figure 3 shows the overall flowchart of the GMP compliant production method of [^18^F]FB-IL2 drug product. The final synthesis methods of [^18^F]SFB and [^18^F]FB-IL2 are described below.

#### Final production process: part 1 - [^18^F]SFB

[^18^F]SFB was produced in three steps using a Zymark robotic system. [^18^F]fluoride was produced by irradiation of [^18^O]water with an IBA cyclotron via the ^18^O(p,n)^18^F nuclear reaction. The aqueous [^18^F]fluoride was passed through a Sep-Pak light QMA anion exchange cartridge (Waters) to recover the ^18^O-enriched water. [^18^F]fluoride was then eluted from the cartridge with 1 mg of potassium carbonate (K_2_CO_3_, Sigma-Aldrich) in 1 mL of water for injections (in-house) and collected in a vial with 5 mg of Kryptofix [2.2.2] (Merck KGaA). To this solution, 1 mL of dry acetonitrile (MeCN, Rathburn) was added and the solvents were evaporated at 130 °C. The radioactive residue ([^18^F]KF - Kryptofix complex) was dried three times by addition and evaporation of anhydrous MeCN (3 × 0.5 mL at 130 °C). After drying, a solution of 10 mg of ethyl 4-(trimethylammonium)benzoate triflate salt (FB precursor, ABX) in 0.25 mL of dry N,N-dimethylformamide (DMF, Sigma-Aldrich) was added and the mixture was allowed to react at 100 °C for 10 min. Then 0.5 mL of 0.3 M sodium hydroxide (NaOH, Merck KGaA) was added and the mixture was allowed to react at room temperature for 5 min. Thereafter, 0.35 mL of 1 M hydrochloric acid (HCl, in-house) was added. The solution was then applied to a C18 light SepPak cartridge (Waters) and washed with 2 × 2 mL of 0.03 M HCl and 2 mL of water for injections. Purified [^18^F]fluorobenzoic acid was eluted from the cartridge with 1 mL of MeCN into a vial containing 10 mg of Kryptofix [2.2.2] and 5 mg of K_2_CO_3_. The eluate was dried under an argon stream at 130 °C. Complete drying was ensured by the addition and evaporation of anhydrous MeCN (3 times 0.5 mL). Then, a solution of 20 mg O-(N-succinimidyl)-1,1,3,3-tetramethyluronium tetrafluoroborate (TSTU, Sigma-Aldrich) in anhydrous MeCN (0.5 mL) was added, and the mixture was heated at 85 °C for 5 min. The mixture was cooled, diluted with 1 M HCl (0.4 mL) and purified by HPLC (Symmetry Shield RP8 5 μm, 7.8 × 300 mm, 40% MeCN in water, flow 4 mL/min). The radioactive product with a retention time of approximately 8 min was collected into a 25 mL sterile vial (Mallinckrodt) via a sterilization filter (Millex-LG filter, 25 mm diameter, 0.2 μm pore size, polytetrafluoroethylene membrane, Millipore). Quality control (QC) was performed, including determination of appearance, yield and radiochemical purity, as described below.

#### Final production process: part 2 - [^18^F]FB-IL2

**Fig. 4:**
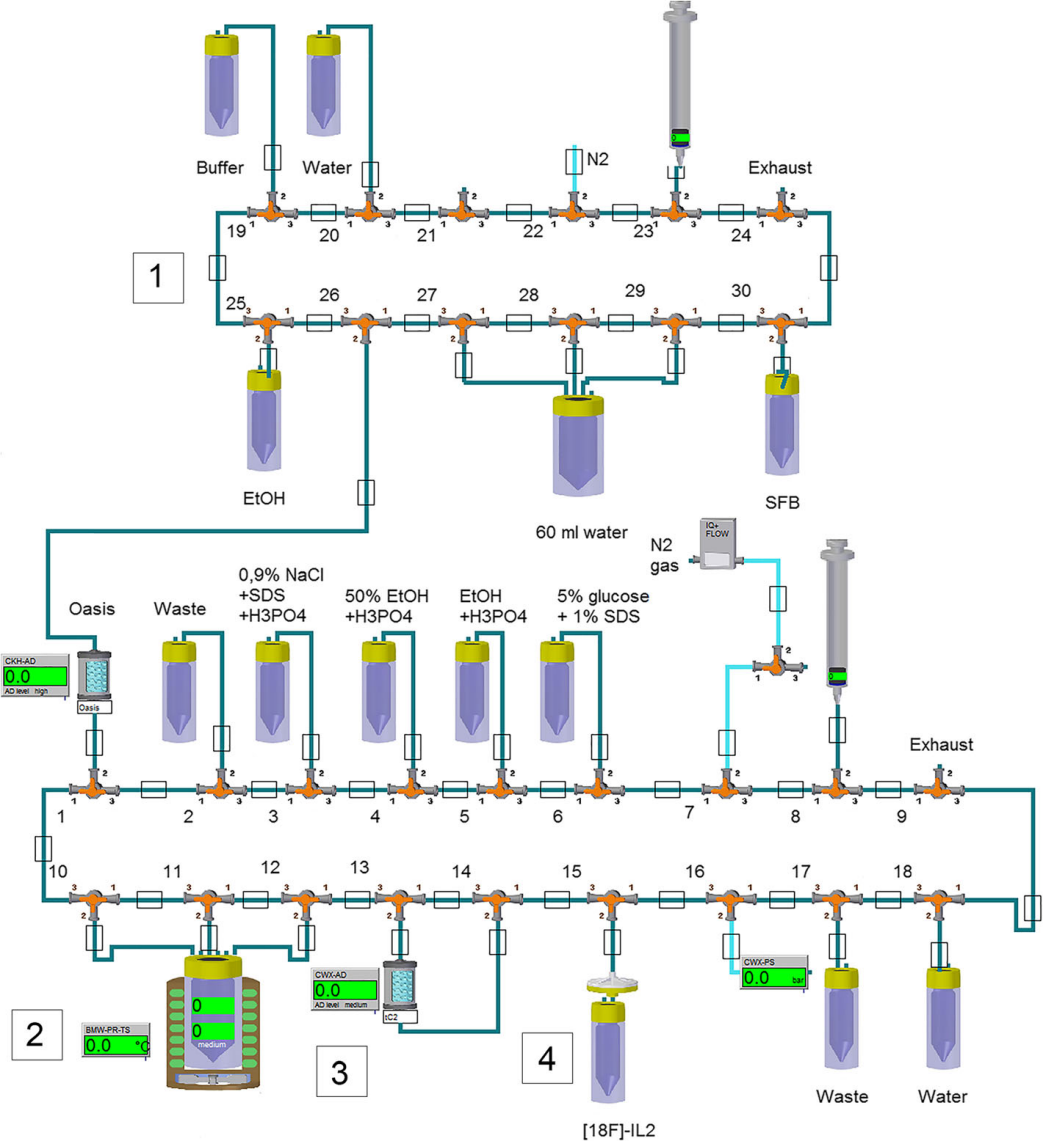
Schematic overview PharmTracer Eckert & Ziegler synthesis module for [^18^F] SFB formulation, followed by [^18^F]FB-IL2 conjugation, purification and filtration. 1) Reformulation [^18^F]SFB using an Oasis HLB cartridge; 2) Conjugation [^18^F]SFB with IL2; 3) Purification [^18^F]FB-IL2 using a tC2 Sep-Pak cartridge; 4) Formulation and sterile filtration of [^18^F]FB-IL2

Figure 4 gives a schematic overview of the set-up of the Modular-Lab PharmTracer Eckert & Ziegler synthesis module used in the second part of the production. This synthesis module is equipped with disposable cassettes (12 valves and 18 valves), polyethylene tubing (Eckert & Ziegler), syringes (Braun) and needles (Becton Dickinson, BD). After QC, [^18^F]SFB was transferred in a lead container to a class C hot cell in a class C cleanroom. The vial containing the [^18^F]SFB in the lead container was connected with a disposable needle and tubing to the synthesis module. With the aid of the syringe of the synthesis module, [^18^F]SFB was transferred from its vial, into 60 ml water for injections. The diluted [^18^F]SFB solution was subsequently passed through an Oasis HLB (1 cc) Sep-Pak cartridge (Waters). The cartridge was washed two times with 5 mL water for injections, dried with a flow of nitrogen gas and eluted with 0.8 mL absolute ethanol (100%, Merck KGaA) into the reactor, which was cooled at − 10 °C and filled with 200 μg of IL2 (Proleukin, 18 × 10^6^ IU), reconstituted in 200 μL water for injections just before the introduction of [^18^F]SFB in the hot cell. Just before elution of [^18^F]SFB, the reactor was set to 50 °C. After collection of the [^18^F]SFB solution in the reactor, 0.8 mL 0.1 M borate buffer (Sigma-Aldrich), pH 8.5, was added. The reaction mixture was heated at 50 °C for 10 min. Thereafter, the reactor was cooled to room temperature and the reaction mixture was diluted with 1 ml sodium chloride 0.9% (Braun) containing 22 μL 25% phosphoric acid (H_3_PO_4_, Sigma-Aldrich) and 48 μL 10% sodium dodecyl sulfate (SDS). After this, the reaction mixture was passed through a tC2 Sep-Pak cartridge (Waters). The reactor and the cartridge were washed three times with 2 mL 50% aqueous ethanol containing 23 μL 25% H_3_PO_4_. The cartridge was then washed with 1 mL of water for injection and thereafter, [^18^F]FB-IL2 was eluted from the cartridge with 1 mL 100% ethanol containing 5 μL 0.25% H_3_PO_4_ and transferred via a sterilization filter (Millex-GV filter, 13 mm diameter, 0.22 μm pore size, Millipore) to a glass vial, European Pharmacopoeia (Ph. Eur.) type I, sterile and pyrogen free, covered with a bromobutyl rubber stopper, sealed with a flip-off aluminum cap (Mallinckrodt/ABX) containing 6.5 mL of 5% glucose, 0.1% SDS and 0.5% human serum albumin (HSA, Albuman, Sanquin) solution. The cartridge and sterilization filter are rinsed with 3.5 mL 5% glucose and 0.1% SDS solution, which is also collected in the sterile vial.

#### Quality control methods

For [^18^F]SFB and the final [^18^F]FB-IL2 drug product quality control (QC) was performed, as shown in Fig. 3. Most QC methods, and their corresponding specifications, are general for radiopharmaceuticals. These methods are compendial methods described in the Ph. Eur, namely tests for osmolarity (Ph. Eur. 2.2.35), residual solvents (acetonitrile, DMF; Ph. Eur. 5.4), bacterial endotoxins (Ph. Eur. 2.6.14). Sterility method is based on Ph. Eur. 2.6.1. A sterility test is performed by adding a sample of the decayed drug product to tryptic soy broth (TBS) medium (Soya-bean casein digest). After 14 days at 25 °C, the clarity of the medium is visually inspected. In case the medium is not clear the sample is tested for the bacterial strain present. Kryptofix is determined by the kryptofix spot test using silica thin-layer chromatography (TLC) strips treated with an aqueous iodoplatinate solution. Discoloration of the strip will be compared with a 25 mg/ml kryptofix reference sample. Radionuclide purity is determined with a germanium detector. Radionuclide identity is determined for the gamma spectrum emitted by the drug product. The half-life is determined by measuring the radioactive decay over time.

Specific for this tracer was the use of ultra-performance liquid chromatography (UPLC) for analysis of (radio) chemical purity, radiochemical identity and molar activity. For this, a Waters Acquity H-Class system and a BEH Shield RP18 column (1.7 μm; 3.0 mm × 50 mm) was used, equipped with both an UV detector (operated at 225 and 280 nm) and a radioactivity detector (Berthold FlowStar LB513, Mx50–6 flow cell). Gradient elution with a mixture of 0.1% aqueous TFA in ultrapure water (solvent A) and 0.1% TFA in mass spectrometry-grade acetonitrile (solvent B) was performed at a flow of 0.8 mL/min. The following gradient profile was used: 0–1 min 5% B, 1–4 min 30% B, 4–6 min 50% B, 6–8 min 50% B, 8–10 min 70% B, 10–11 min 5% B. Retention times were 3.9 min for [^18^F]fluorobenzoic acid ([^18^F]FBA), 4.9 min for [^18^F]SFB, 5.6 min for HSA and 9.0 min for [^18^F]FB-IL2.

### Validation

#### Validation of analytical methods

In order to produce a radiopharmaceutical according to GMP regulations, QC with validated analytical methods is needed. Validation of compendial tests for osmolarity, residual solvents, endotoxins and sterility was conducted previously according to the respective compendial monographs, as applicable. However, the specific UPLC method for the QC of [^18^F]FB-IL2 needed to be validated. Additional file 1: Table S1 describes the different tests for the validation of the UPLC QC method and their corresponding acceptance criteria.

#### Validation of the production method

To assure that the production method is robust and results in a product with consistent quality, validation of the production method was performed. Validation of [^18^F]FB-IL2 consisted of four independent productions, including QC, as shown in the results section. All batch productions had to comply with the predefined specifications summarized in Table 2. During validation the stability of [^18^F]FB-IL2 has been investigated using UPLC analysis. Radiochemical purity was determined directly after labeling and 1 h after production.

### Documentation

Documentation is an essential part in the development of a radiopharmaceutical produced according to GMP regulations and is needed to prove the overall quality of the final product (Patel & Chotai, 2011). All methods are documented in Standard Operation Procedures (SOPs). Validation results of both analytical methods and the production method are documented in performance qualification (PQ) validation reports, which are authorized by a Qualified Person (QP). The Master Batch Record (MBR) is drafted to describe the general production process, including details on reagents, materials and equipment used, and specific step-by-step instructions for production. Test methods are provided with instructions for testing supplies, materials, products, and other production-related tasks and activities (Patel & Chotai, 2011). The Investigational Medicinal Product Dossiers (IMPD) is drafted according to EU guidelines (Committee for Medicinal Products for Human Use, 2017; Todde et al., 2014).

## Results

### Technology transfer

Technology transfer describes the translation of methods developed in R&D setting to a GMP environment (Fig. 1). This environment can potentially differ in terms of equipment, material and personnel. Moreover, production needs to be fully automated and performed in a closed and shielded hot cell. To avoid cross-contamination, synthesis modules with disposable cassettes are recommended over modules with fixed tubing (otherwise, thorough validation of washing procedures would be required) or manual or robotic methods. To ensure sterility of the final product the production needs to be performed in a classified cleanroom and hot cell, with the final filtration step in grade A in B. Additionally the final drug product needs to be of consistent predefined quality, stable and in a formulation suitable for clinical application. The initial transfer of the production of both [^18^F]SFB and [^18^F]FB-IL2 to the Eckert & Ziegler synthesis module led to several issues. Table 1 shows those issues and the adaptations that had to be made.

**Table 1:** Challenges encountered during translation from research grade to clinical grade [^18^F]FB-IL2 and adaptations made to the original preclinical protocol

Preclinical production method	Challenge encountered	GMP-production method
Part 1: [^18^F]SFB synthesis		
Purification by Oasis HLB cartridge.	Impurity in [^18^F]SFB product interfered with the conjugation of IL2.	Purification performed by HPLC, followed by formulation using an Oasis HLB cartridge.
[^18^F]SFB is produced in a non-classified hot cell with a Zymark robotic system. [^18^F]FB-IL2 produced manually.	GMP syntheses modules insufficient functionalities to accommodate the total labeling procedure, due to a change in purification method.	[^18^F]SFB produced as a starting material in a non-classified hot cell with a Zymark robotic system. Product collected via sterile filtration in sterile vial and transferred to a class C clean room. [^18^F]FB-IL2 produced with a PharmTracer Eckert & Ziegler synthesis module in a class C clean room and class C hot cell.
Part 2: [^18^F]FB-IL2 conjugation		
Reconstituted IL2 stored at −80 °C. After defrosting added manually to the mixture immediately before conjugation.	IL2 labeling in automated synthesis module: IL2 added at start of synthesis. IL2 instable in warm hot cell during synthesis.	After production of [^18^F]SFB IL2 added, directly to cooled reaction vial. Temperature to 50 °C shortly before adding [^18^F]SFB.
	IL2 transported via disposable tubing to the reaction vial. Sticking of IL2 to tubing during transport to the reaction vial.	
Conjugation reaction in borate buffer pH 8.3.	Low yields due to low labeling efficiencies. At pH > 8.7 hydrolysis of [^18^F]SFB.	Optimal pH 8.5 (±0.1).
HPLC purification after conjugation step.	Low recovery [^18^F]FB-IL2 from HPLC due to high lipophilicity and tendency to aggregate.	HPLC replaced by solid-phase extraction over a tC2 Sep-Pak cartridge.
No sterile filtration for animal experiments.	[^18^F]FB-IL2 tendency to stick to sterilization filter.	Filter size changed to smaller diameter.
End formulation in ~ 55% ethanol in water, diluted 1:10 with saline (ethanol < 10%) shortly before use.	Final formulation adapted for patient use: 9% ethanol, 4.5% glucose, 0.5% HSA, 0.1% SDS and 0.0001% phosphoric acid. An excipient in the final formulation occasionally precipitated.	Precipitation of HSA at low pH (< 4). Amount of phosphoric acid at washing/elution step adapted (100 times diluted).

Abbreviations: *HLB* hydrophilic-lipophilic balance, *IL2* interleukin-2, *HPLC* high performance liquid chromatography, *GMP* good manufacturing practices, *HSA* human serum albumin, *SDS* sodium dodecyl sulfate

#### Part 1 - [^18^F]SFB

The initial setup for the GMP compliant production of [^18^F]FB-IL2 resulted in low yields and frequent failures. An important cause for these disappointing results was an inefficient purification of [^18^F]SFB by solid-phase extraction with an Oasis HLB Sep-Pak cartridge, resulting in an impurity in the starting material for the conjugation. This impurity appeared to compete with [^18^F]SFB for the binding sites of IL2 (the primary amino group of lysine residues), resulting in low yields. Liquid chromatography–mass spectrometry (LC-MS) and UPLC were performed to characterize the impurity. These analyses showed that the impurity had a molecular weight (301 g/mol) equal to that of TSTU, which is used as a reagent in the [^18^F]SFB synthesis. However, the retention time on UPLC was distinctly different from that of TSTU. We have not been able to elucidate the identity of the impurity. To improve separation of this unidentified impurity from [^18^F]SFB we replaced the solid phase extraction method by a preparative HPLC method using a reversed-phase Symmetry Shield column with 40% MeCN in water as the eluent. With this method, the interfering impurity could be adequately separated from [^18^F]SFB. Due to the large volume of the HPLC fraction containing [^18^F]SFB and the presence of MeCN in the eluent, the collected product had to be reformulated before it could be used in the conjugation reaction with IL2. [^18^F]SFB was reformulated in a small volume of ethanol by solid phase extraction with an Oasis HLB (1 cc) Sep-Pak.

As a result of the modification of the [^18^F]SFB purification procedure, the Eckert & Ziegler synthesis modules had insufficient functionalities to accommodate the complete labeling procedure. As the optimized purification method could not be implemented in one single module, the [^18^F]SFB production was separated from the conjugation procedure. [^18^F]SFB was considered as a starting material, rather than an intermediate, and its production was performed in a non-classified hot cell with a Zymark robotic system. HPLC-purified and sterile filtrated [^18^F]SFB was subjected to the quality control procedure and transferred to a class C cleanroom and a class C hot cell. There, the procedure started with the reformulation step using the Modular-Lab PharmTracer Eckert & Ziegler synthesis module.

#### Part 2 - [^18^F]FB-IL2

Another cause for low yields during the conjugation reaction was the instability of IL2. At room temperature, IL2 has the tendency to aggregate after reconstitution in water. Moreover, at higher temperatures (> 60 °C) the protein rapidly denatures (Di Gialleonardo et al., 2012b). During preclinical development, the protein solution was therefore collected from storage just before the start of the production and kept at − 10 °C until the start of the reaction with [^18^F]SFB. For preclinical studies, the reconstituted IL2 could be added manually shortly before the conjugation reaction. However, this was not an option for the clinical production, since the production was performed with an automated synthesis module in a closed hot cell. A reagent vial with the protein solution already had to be connected to the cassette in the synthesis module at the start of the [^18^F]SFB synthesis. Since the IL-2 solution was not cooled, this resulted in aggregation of the protein in the warm hot cell (approximately 2 h at > 30 °C). An external cooling device was therefore implemented to keep the IL2 reagent vial cold. Unfortunately, this did not result in a higher yield. A possible explanation for this disappointing result was that during transport via tubing from the external cooling device to the reaction vial IL2 adsorbed to the long disposable polyethylene tubing and valves. As for the conjugation reaction only 200 μL of IL2 solution is used, a relatively large fraction of the solution was adsorbed to the long tubing. To overcome this problem, the IL2 solution was added directly to the reaction vial, which was cooled at − 10 °C in a Peltier heating and cooling system until the start of the conjugation reaction. Just before the [^18^F]SFB solution was transferred to this reaction vial, the temperature of the oven was increased to the reaction temperature of 50 °C.

Another critical parameter in the conjugation was the narrow pH range of the reaction mixture required for the conjugation reaction. Previously the conjugation reaction was performed in borate buffer pH 8.3. We found higher yields in a borate buffer pH 8.5. Higher pH values (> 8.5) led to faster hydrolysis of [^18^F]SFB, reducing the amount of SFB available for conjugation to IL2. And at lower pH values, a larger fraction of the amino groups of the lysine residues of the protein is protonated and thus not readily available for the reaction with [^18^F]SFB.

As mentioned above, IL2 has the tendency to aggregate and to adhere to tubing, valves, HPLC columns and sterilization filters. As a result, the recovery of [^18^F]FB-IL2 from the HPLC system was poor. Therefore, the HPLC method was replaced by a solid-phase extraction using a tC2 Sep-Pak cartridge. With this method the surface area for the protein to adhere to is lower, resulting in a higher recovery. This method has previously been used for the purification of ^99m^Tc-HYNIC-IL2 (D’Alessandria et al., 2010), and also proved suitable for the purification of [^18^F]FB-IL2. The expected impurities, namely [^18^F] fluoride, [^18^F]FBA and unreacted [^18^F]SFB, were first washed from the tC2 Sep-Pak cartridge with an acidified 50% aqueous ethanol solution (pH = 1). Thereafter, [^18^F]FB-IL2 was washed from the column with a more apolar eluent (acidified 100% ethanol), thereby also reducing adsorption to tubing and valves. When using the standard low protein binding sterilization filters (diameter 33 mm), still approximately 50% of the radiolabeled product adsorbed to the filter. When filters with a smaller diameter size (diameter 13 mm) were used, the amount of protein that adsorbed to the filter was substantially reduced to 25%.

To further prevent aggregation and adsorption, HSA, glucose and SDS were added to the final formulation. The pH of this final formulation was critical, as we occasionally observed precipitation of a white substance in the product vial. This precipitation was reversible and appeared to be pH dependent as it dissolved again by increasing the pH using sodium hydroxide (NaOH). Therefore, the concentration of H_3_PO_4_, which was used during elution of the tC2 Sep-Pak cartridge, was reduced from 5 μL 25% H_3_PO_4_ in 1 mL ethanol to 5 μL 0.25% H_3_PO_4_ in 1 mL ethanol and by an additional washing of the cartridge with water for injection to remove residual acid from the cartridge, valves and tubing before elution of the product. The final formulation for patient use contains 9% ethanol, 4.5% glucose, 0.5% HSA, 0.1% SDS and 0.0001% H_3_PO_4._

### Validation

#### Validation of analytical methods

All PQ test results for the UPLC method validation were within acceptance criteria (Additional file 1: Table S1). Some specific important issues for the analysis of [^18^F]FB-IL2 using UPLC had to be taken into account. First, addition of HSA resulted in a large UV peak in the UPLC chromatogram. However, the resolution between HSA and other components is sufficient (> 1.5) to distinguish the HSA peak from the other components. Second, the protein IL2 could be detected at a wavelength of 280 nm. However, the UV absorption of some impurities at 280 nm was insufficient to be detected. These impurities could be adequately measured at 225 nm and therefore a dual wavelength detector, operated simultaneously at 225 and 280 nm, was used. Third, the maximum concentration of IL2 in the tracer solution is close to the detection limit. For conditioning of the UPLC column it was important to perform two runs with matrix (formulation buffer) before injection of the radioactive sample. Otherwise carry-over of impurities of previous runs could interfere with the analysis.

Finally, radiolabeled IL2 is slightly more lipophilic than unconjugated IL2. A free amino group, which is protonated (and therefore positively charged) under physiological conditions, is converted into a neutral amide group after the conjugation reaction. This resulted in a delay in retention time of 0.6 to 1.0 min, as compared to naïve IL2.

#### Validation of the production method

Validation results are displayed in Table 2. [^18^F]FB-IL2 could be produced with a consistent quality complying to all predefined specifications as described in Table 2. The radiochemical yield of [^18^F]FB-IL2 was 3–4%, based on the amount of [^18^F]SFB used. Despite the low radiochemical yield, one production yields sufficient radiotracer for injection of one or two patients. Stability was investigated by measuring the radiochemical purity directly after labeling and 1 h after production. Table 3 shows that within 1 h the radiochemical purity did not decrease more than 0.3%. This was within the test-retest variability of the UPLC analysis method. Therefore, the expiration time is set at 1 h.

**Table 2:** QC specifications and validation results of [^18^F]SFB and [^18^F]FB-IL2 synthesis

Test	Specification	Justification	Batch 1	Batch 2	Batch 3	Batch 4
[^18^F]SFB						
Appearance	Clear, colorless	Ph. Eur.	clear, colorless	clear, colorless	clear, colorless	clear, colorless
Minimum yield	> 2 GBq	Minimum amount required to produce a patient dose of [^18^F]FB-IL2	20.2 GBq	19 GBq	19.5 GBq	12.4 GBq
Radiochemical purity	> 90%	Adequate purity for a starting material in the conjugation reaction	96.0%	94.9%	95.7%	99.9%
[^18^F]FB-IL2						
Appearance	Clear, colorless	Ph. Eur.	clear, colorless	clear, colorless	clear, colorless	clear, colorless
PH	4–7	Ph. Eur	4.5	5	4.5	7
Radiochemical identity:		Standard requirement				
- retention time IL2 (UPLC)	- ca. 9 min		- 8.9 min	- 9.0 min	- 8.9 min	- 9.0 min
- retention time [^18^F]FB-IL2 (UPLC)	- IL2 standard + 0.6–1.0 min		- 9.6 min	- 9.7 min	- 9.8 min	- 9.5 min
Radiochemical purity	> 95%	Ph. Eur.	97.1%	97.4%	95.7%	98.0%
Radionuclide purity	> 99%	Ph. Eur.	> 99%	> 99%	> 99%	> 99%
Radionuclide identity	T_1/2_ 110 min; acceptable range 105–115 min	Ph. Eur.	115 min	115 min	112 min	112 min
	Energy 511 keV		511 keV	511 keV	511 keV	511 keV
[^18^F]FB-IL2 concentration	< 5 mg/L	Corresponds to a max dose of 50 μg (3 nmol) for a standard injection volume of 10 mL	< 3.7 mg/L^1^	< 3.7 mg/L	< 3.7 mg/L	< 3.7 mg/L
Unknown impurities	< 1 mg/L		< 1 mg/L	< 1 mg/L	- < 1 mg/L	< 1 mg/L
Molar activity	> 50,000 MBq/μmol	Corresponds with mass dose [^18^F]FB-IL2 of 50 μg at an injected dose of 200 MBq	> 50,000 MBq/μmol	> 50,000 MBq/μmol	> 50,000 MBq/μmol	> 50,000 MBq/μmol
Filter integrity (pressure hold test/bubble point test)	> 3.4 bar	Conforms to filter manufacturer’s recommendations	> 3.4 bar	> 3.4 bar	> 3.4 bar	> 3.4 bar
Sterility	Sterile^2^	Ph. Eur. 2.6.1	sterile	sterile	sterile	sterile
Endotoxins	< 2.5 EU/mL	Ph. Eur. 2.6.14	< 0.5 EU/mL	< 0.5 EU/mL	< 0.5 EU/mL	< 1.0 EU/mL
Kryptofix 222	< 25 mg/L	Ph. Eur.	< 25 mg/L	< 25 mg/L	< 25 mg/L	< 25 mg/L
Osmolarity	< 3000 mOsmol/kg^2^	Ph. Eur. 2.2.35 Based on formulation	2760 mosmol/kg	2940 mosmol/kg	2583 mosmol/kg	2790 mosmol/kg
Acetonitrile	< 410 mg/L^2^	Ph. Eur. 2.4.24	< 10 mg/L	< 10 mg/L	< 10 mg/L	< 10 mg/L
DMF	< 880 mg/L^2^	Ph. Eur. 2.4.24	< 200 mg/L	< 200 mg/L	< 200 mg/L	< 200 mg/L
Ethanol	< 150 g/L^2^	Based on formulation	87.7 g/L	102.7 g/L	88.0 g/L	105 g/L

^1^Lower limit of detection = 3.7 mg/L; ^2^Post-release test. Abbreviations: *QC* Quality control, *UPLC* ultra-performance liquid chromatography, *DMF* dimethylformamide

**Table 3:** Stability data of [^18^F]FB-IL2

Batch	Radiochemical purity (%)	
	T = 0	T = 1 h
1	97.1%	97.4%
2	97.4%	97.1%
3	95.7%	96.2%
4	98.0%	99.0%

### Documentation

As described in the methods section, all methods and the production process have been documented to ensure traceability of the production of [^18^F]FB-IL2. The IMPD has been written, which includes information regarding quality and production of [^18^F]FB-IL2, including all results of validation. The IMPD also included an Investigator’s Brochure (IB) part on pharmacology, pharmacokinetics and toxicology.

Previous radiolabeled IL2 tracers have shown to be safe for human use. Preclinical studies revealed that in [^18^F]FB-IL2, the perturbation of the protein by the small label [^18^F] FB are minimal and consequently the tracer has a similar in vivo behavior as recombinant human IL2. Moreover, the tracer was administered in a sub-pharmacological dose range (≤50 μg, therapeutic dose is 1.1 mg). According to the ICH guideline M3(R2) this is in the micro dose range (Committee for Medicinal Products for Human Use, 2009). For these reasons it is justified that no additional toxicity studies have been performed for [^18^F]FB-IL2. Besides, interpretation of the results of toxicity studies of a human protein in rodents would be highly complicated, as the results could be false negative due to species differences in biological activity, or false positive due to an immune response to the foreign protein. This rationale is also described in the IMPD, section 2.2.4. The IMPD has been approved by both the Dutch national competent authorities (CCMO18.0508) and the institutional medical ethics review board (METc2014/373; METc207/174; METc2017/202 and METc2017/418) and is included as Additional file 2.

## Discussion

The road to a radiopharmaceutical suitable for clinical studies can be challenging and time-consuming. Major barriers and unexpected problems might be encountered during the translation from a R&D to a GMP production method. Here we exemplify this road with the complex production method of [^18^F]FB-IL2, the first-in-human fluor-18 labeled IL2 tracer for use in clinical studies to image activated T-cells. This radiopharmaceutical can now be produced consistently, although still in relatively low yields, sufficient for one or two patients. The final product meets the strict GMP requirements and is safe to use in clinical studies. We hope the description of this complex translation, including IMDP, contributes to a faster development of other new interesting radiopharmaceuticals.

There is a great interest in molecular imaging of activated T-cells. To further improve the production of this promising radiopharmaceutical the potential causes for the still relatively low yields have to be further investigated in the future. During synthesis, small volumes were being transported via relatively long tubing. This could lead to a loss of tracer, especially since IL2 has the tendency to adhere to tubing. Moreover, only a very low amount of protein was used, as IL2 has already pharmacological effects at a low concentration. Finally, during purification with the tC2 cartridge, part of the radiolabeled IL2 might have been washed away with the 50% aqueous ethanol solution. Some solutions to these problems have been described, for instance increasing the stability of the protein by adding SDS to the final formulation. This resulted in a final formulation which was safe for human use, but with a high osmolality. Therefore, the tracer should be injected via a slow bolus injection. Other ways to further improve the yield will be investigated in the future and increase availability for larger amounts of patients.

Although we are now able to produce this tracer consistently, there are still some challenges left. The current production method is time-consuming and labor-intensive. For the future other radiolabeling methods, for instance chelation of radiometal PET isotopes such as copper-64, gallium-68, zirconium-89 or aluminum [^18^F]fluoride, will be investigated. Given the biological half-life of IL2 (around 100 min), the most suitable radiometals in this context are likely either gallium-68 or aluminum [^18^F]fluoride.

## Conclusions

[^18^F]FB-IL2 is an interesting tracer for T-cell imaging that warrants clinical proof of concept testing. To be able to do so a clinical grade radiopharmaceutical is a prerequisite. The partly manual, preclinical production method was translated to a GMP environment. This translation led to challenges in automation, purification, formulation and stability. However, we are now able to produce [^18^F]FB-IL2 consistently and have this innovative tracer available for use in clinical studies.

## Additional files

Additional file 1: **Table S1.** Performance qualification tests for the quality control of [^18^F]FB-IL2 using UPLC. (DOCX 17 kb)

Additional file 2: Investigational medicinal product dossier: [^18^F]FB-IL2. (DOC 938 kb)

## Data Availability

The data generated during the current study are available from the corresponding author on reasonable request.

## Abbreviations

[^18^F]SFB: N-succinimidyl 4-[^18^F]fluorobenzoate
DMF: N,N-dimethylformamide
FBA: Fluorobenzoic acid
GC: Gas chromatography
GMP: Good manufacturing practices
H_3_PO_4_: Phosphoric acid
HCl: Hydrochloric acid
HLB: Hydrophilic-lipophilic balance
HPLC: High performance liquid chromatography
HSA: Human serum albumin
IL2: Interleukin-2
IMPD: Investigational medicinal product dossier
K_2_CO_3_: Potassium carbonate
LAL: Limulus amebocyte lysate
MBR: Master batch record
MeCN: Acetonitrile
NaOH: Sodium hydroxide
PET: Positron emission tomography
Ph. Eur: European Pharmacopoeia
PQ: Performance qualification
QC: Quality control
R&D: Research and development
SDS: Sodium dodecyl sulfate
SOP: Standard operation procedures
SPECT: Single photon emission computed tomography
TFA: Trifluoroacetic acid
TLC: Thin-layer chromatography
UPLC: Ultra-performance liquid chromatography
UV: Ultraviolet
TSTU: O-(N-succinimidyl)-1,1,3,3-tetramethyluronium tetrafluoroborate
[^18^F]FB-IL2: N-(4-[^18^F]fluorobenzoyl)-interleukin-2
