# Supplementary material for: Clinical-grade N-(4-[18F]fluorobenzoyl)-interleukin-2 for PET imaging of activated T-cells in humans
Source: EJNMMI Radiopharm Chem. 2019 Jul 17;4:15. doi: 10.1186/s41181-019-0062-7 (PMC6637160; doi:10.1186/s41181-019-0062-7)
Supplement: Supplementary file 1 — Table S1. Performance qualification tests for the quality control of [18F]FB-IL2 using UPLC. (DOCX 17 kb) [file 41181_2019_62_MOESM1_ESM.docx]

**ADDITIONAL FILE 1**

**Table S1** Performance qualification tests for the quality control of [^18^F]FB-IL2 using UPLC

| Test | Description | Acceptance criteria | Results validation (average ± SD) |
| --- | --- | --- | --- |
| Retention times | Retention times of the different components measured at three different days. | <10% difference. | FBA: 3.9 ± 0.0  SFB: 4.9 ± 0.0  HSA: 5.6 ± 0.1  IL2: 9.0 ± 0.1 |
| Linearity UV signal | Calibration curves of different components measured on 3 different days. | <5% difference in the slope.  R^2^ > 0.98 | difference FBA = 2.0% ± 0.9%  difference SFB = 4.0% ± 3.4%  difference HSA = 1.0% ± 0.8%  difference IL2 = 2.4% ± 1.3%  R^2^ FBA: 0.9998 ± 0.0001  R^2^ SFB: 0.9967 ± 0.0012  R^2^ HSA: 0.9993 ± 0.0001  R^2^ IL2: 0.9987 ± 0.0006 |
| Precision | Concentrations of reference standards measured 5 times. | <5% difference. | FBA: 1.3%  SFB: 0.6%  HSA: 1.8%  IL2: 1.5% |
| Carry-over | High concentration reference standard injected, followed by a matrix injection (formulation buffer: ethanol, glucose, SDS solution). | <0.1% carry-over. | FBA: 0%  SFB: 0%  HSA: 0.06%  IL2: 0% |
| Recovery [^18^F]FB-IL2 | Radioactivity in the injection sample measured before injection. The eluted solvent is collected and the total eluted radioactivity is measured. | <10% difference. | Difference 4.9% ± 1.9% |
| LOD and LOQ IL2 | Noise determined on chromatogram of sample or calibration curve | LOD = 3x noise, LOQ = 10x noise. | LOD concentration = 1.1 mg/L  LOQ concentration = 3.7 mg/L |
| Peak resolution | Low concentration of reference standards measured and differences in retention times analyzed. | Resolution>1.5. | Resolution FBA – SFB = 3.7  Resolution SFB – HSA = 2.5  Resolution HSA – IL2 = 7.7 |
| Reproducibility | Lowest, middle and high concentration in calibration curve measured five times on three different days. | <5% difference. | Lowest = 0.5% ± 0.4% difference.  Middle = 0.8% ± 0.4% between days.  High = 0.5% ± 0.4% |
| Matrix signal | Matrix injected three times. | No peaks higher than 3x noise besides known compounds. | No peaks higher than 3x noise. |

Abbreviations: UV: ultraviolet; SDS: sodium dodecyl sulfate; LOD: limit of detection; LOQ: limit of quantification; FBA: fluorobenzoic acid; SFB: N-succinimidyl 4-fluorobenzoate; HSA: human serum albumin
