# Supplementary material for: Clinical-grade N-(4-[18F]fluorobenzoyl)-interleukin-2 for PET imaging of activated T-cells in humans
Source: EJNMMI Radiopharm Chem. 2019 Jul 17;4:15. doi: 10.1186/s41181-019-0062-7 (PMC6637160; doi:10.1186/s41181-019-0062-7)
Supplement: Supplementary file 2 — Investigational medicinal product dossier: [18F]FB-IL2. (DOC 938 kb) [file 41181_2019_62_MOESM2_ESM.doc]

**INVESTIGATIONAL MEDICINAL PRODUCT DOSSIER**

**[^18^F]FB-IL2**

**TABLE OF CONTENTS**

[2. CHEMICAL PHARMACEUTICAL AND BIOLOGICAL DATA 5](#__RefHeading___Toc515599219)

[2.1 CHEMICAL PHARMACEUTICAL DATA 5](#__RefHeading___Toc515599220)

[2.1.S DRUG SUBSTANCE 5](#__RefHeading___Toc515599221)

[2.1.S.1 General information 5](#__RefHeading___Toc515599222)

[2.1.S.1.1 Nomenclature 5](#__RefHeading___Toc515599223)

[2.1.S.1.2 Structure 5](#__RefHeading___Toc515599224)

[2.1.S.1.3 General Properties 5](#__RefHeading___Toc515599225)

[2.1.S.2 Manufacture 6](#__RefHeading___Toc515599226)

[2.1.S.2.1 Manufacturer(s) 6](#__RefHeading___Toc515599227)

[2.1.S.2.2 Description of Manufacturing Process and Process Controls 7](#__RefHeading___Toc515599228)

[2.1.S.2.4 Controls of Critical Steps and Intermediates 8](#__RefHeading___Toc515599229)

[2.1.S.2.5 Process validation and/or Evaluation 9](#__RefHeading___Toc515599230)

[2.1.S.2.6 Manufacturing Process Development 9](#__RefHeading___Toc515599231)

[2.1.S.3 Characterization 9](#__RefHeading___Toc515599232)

[2.1.S.3.1 Elucidation of Structure and Other Characteristics: 9](#__RefHeading___Toc515599233)

[2.1.S.4 Control of Drug Substance 12](#__RefHeading___Toc515599234)

[2.1.S.4.1 Specification 12](#__RefHeading___Toc515599235)

[2.1.S.4.2 Analytical Procedures 12](#__RefHeading___Toc515599236)

[2.1.S.4.3 Validation of Analytical Procedures. 12](#__RefHeading___Toc515599237)

[2.1.S.4.4 Batch Analyses 12](#__RefHeading___Toc515599238)

[2.1.S.4.5 Justification of specifications for drug substance 12](#__RefHeading___Toc515599239)

[2.1.S.5 Reference Standards or Materials 12](#__RefHeading___Toc515599240)

[2.1.S.6 Container Closure System 12](#__RefHeading___Toc515599241)

[2.1.S.7 Stability 12](#__RefHeading___Toc515599242)

[2.1.P MEDICINAL PRODUCT 13](#__RefHeading___Toc515599243)

[2.1.P.1 Description and Composition of the DRUG Product 13](#__RefHeading___Toc515599244)

[2.1.P.2 Pharmaceutical development 14](#__RefHeading___Toc515599245)

[2.1.P.2.1 Components of the Medicinal Product 14](#__RefHeading___Toc515599246)

[2.1.P.2.2 Medicinal product 14](#__RefHeading___Toc515599247)

[2.1.P.2.3 Manufacturing process development 14](#__RefHeading___Toc515599248)

[2.1.P.2.4 Container Closure System 14](#__RefHeading___Toc515599249)

[2.1.P.2.5 Microbiological Attributes 14](#__RefHeading___Toc515599250)

[2.1.P.2.6 Compatibility 14](#__RefHeading___Toc515599251)

[2.1.P.3 Manufacture 15](#__RefHeading___Toc515599252)

[2.1.P.3.1 Manufacturer 15](#__RefHeading___Toc515599253)

[2.1.P.3.2 Batch Formula 15](#__RefHeading___Toc515599254)

[2.1.P.3.3 Description of manufacturing process 15](#__RefHeading___Toc515599255)

[2.1.P.3.4 Control of critical steps and intermediates 16](#__RefHeading___Toc515599256)

[2.1.P.3.5 Process Validation and/ or Evaluation 16](#__RefHeading___Toc515599257)

[2.1.P.4 Control of Excipients 16](#__RefHeading___Toc515599258)

[2.1.P.4.1 Specifications 16](#__RefHeading___Toc515599259)

[2.1.P.4.2 Analytical procedures 16](#__RefHeading___Toc515599260)

[2.1.P.4.3 Validation of Analytical Procedures 16](#__RefHeading___Toc515599261)

[2.1.P.4.4 Justification of Specifications 16](#__RefHeading___Toc515599262)

[2.1.P.4.5 Excipients of Human or Animal Origin 16](#__RefHeading___Toc515599263)

[2.1.P.4.6 Novel Excipients 17](#__RefHeading___Toc515599264)

[2.1.P.5 Control of DRUG product 17](#__RefHeading___Toc515599265)

[2.1.P.5.1 Specifications 17](#__RefHeading___Toc515599266)

[2.1.P.5.2 Analytical Procedures 18](#__RefHeading___Toc515599267)

[2.1.P.5.3 Validation of Analytical Procedures 19](#__RefHeading___Toc515599268)

[2.1.P.5.4 Batch Analyses 20](#__RefHeading___Toc515599269)

[2.1.P.5.5 Characterization of impurities 20](#__RefHeading___Toc515599270)

[2.1.P.5.6 Justification of Specifications 21](#__RefHeading___Toc515599271)

[2.1.P.5.7 Reference standards 23](#__RefHeading___Toc515599272)

[2.1.P.6 Container Closure System 23](#__RefHeading___Toc515599273)

[2.1.P.7 Stability 23](#__RefHeading___Toc515599274)

[2.2 Non-clinical pharmacology, pharmacokinetics and toxicology 23](#__RefHeading___Toc515599275)

[2.2.1 Non-clinical pharmacology 24](#__RefHeading___Toc515599276)

[2.2.2 Non-clinical pharmacokinetics 27](#__RefHeading___Toc515599277)

[2.2.3 Test Materials used in toxicity studies 28](#__RefHeading___Toc515599278)

[2.2.4 Integrated assessment of the data package 28](#__RefHeading___Toc515599279)

[2.2.5 List of studies Conducted & References 28](#__RefHeading___Toc515599280)

[2.2.6 GLP Statement and bioanalytical methods 29](#__RefHeading___Toc515599281)

[2.3 CLINICAL DATA 29](#__RefHeading___Toc515599282)

[2.3.1 Clinical pharmacology 29](#__RefHeading___Toc515599283)

[2.3.2. Clinical pharmacokinetics 29](#__RefHeading___Toc515599284)

[2.3.4 Human exposure 30](#__RefHeading___Toc515599285)

[2.4 Overall risk and benefit assessment 31](#__RefHeading___Toc515599286)

Table of figures

Figure 1 Structure of [^18^F]FB-IL2 (relative size of the protein is strongly reduced)) [5](#__RefHeading___Toc517788635)

Figure 2 Schematic of the radiosynthesis of [18F]FB-IL2, consisting of 2 steps: a) preparation of the activated ester [18F]SFB and b) conjugation of [18F]SFB to IL2. [7](#__RefHeading___Toc517788636)

Figure 3: UPLC chromatogram of Proleukin® (UV signal, blue line) and [^18^F]FB-IL2 (radioactivity signal, black line). [9](#__RefHeading___Toc517788637)

Figure 4: Characterization of [^18^F]FB-IL2 by MALDI-ToF [10](#__RefHeading___Toc517788638)

Figure 5:Gel electrophoresis of Proleukin® (lane 1), unpurified reaction mixture of IL2 labeling (lane 2) and purified [18F]FB-IL2 (lane 3 and 4). The Coomassie Brilliant Blue image is superimposed on the autoradiogram of the gel. [10](#__RefHeading___Toc517788639)

Figure 6: MTT assay on PHA-activated PBMCs stimulated by either native IL2 or decay [^18^F]FB-IL2. [11](#__RefHeading___Toc517788640)

Figure 7: TCA Precipitation test of [^18^F]FB-IL-2 in PBS and human plasma [13](#__RefHeading___Toc517788641)

Figure 8:Ex-vivo biodistribution of [^18^F]FB-IL2 in healthy immune-competent Balb/c mice [24](#__RefHeading___Toc517788642)

Figure 9:A) Transaxial PET image, showing the tracer uptake at the injection site of human PBMCs in SCID mice (red arrow) and the migration of PBMC to the contra-lateral side (yellow arrow), due to a local inflammation. B) The effect of pretreatment with a 100-fold excess Proleukin® (Blocked) on the uptake of [^18^F]FB-IL2 in the PBMC xenograft. [25](#__RefHeading___Toc517788643)

Figure 10:Coronal (A) and transaxial (B) [18F]FB-IL2 PET images of a diabetic-prone BB/W rat, showing high uptake in the infiltrated pancreas (left). Tracer uptake was highly dependent on the extent of insulitis (i.e. the amount of infiltrating T lymphocytes) (right) [26](#__RefHeading___Toc517788644)

Figure 11:Time-activity curves of [18F]FB-IL2 in plasma (left) and a PBMC xenograft (right) in Wistar rats. [27](#__RefHeading___Toc517788645)

**Table of tables**

[Table 1: List of materials used in a [^18^F]FB-IL2 batch production 8](#__RefHeading___Toc517788646)

[Table 2: Requirements for the intermediate [^18^F]SFB 8](#__RefHeading___Toc517788647)

[Table 3: Composition of a Batch of Drug Product [^18F^]FB-IL2 (approximately 10 ml) 13](#__RefHeading___Toc517788648)

[Table 4: Batch formula of [^18^F]FB-IL2 15](#__RefHeading___Toc517788649)

[Table 5: List of excipients in the [^18^F]FB-IL2 drug product solution 16](#__RefHeading___Toc517788650)

[Table 6: Specifications of [^18^F]FB-IL2 17](#__RefHeading___Toc517788651)

[Table 9:List of non-clinical studies for [^18^F]BF-IL2 28](#__RefHeading___Toc517788652)

[Table 10:Rate of clearance of 99mTc-IL2 from different organs and tissues (minutes) [10]. 30](#__RefHeading___Toc517788653)

## 1. INTRODUCTION

The investigational radiopharmaceutical is N-(4-[^18^F]fluorobenzoyl)interleukin-2 ([^18^F]FB-IL2). [^18^F]FB-IL2 is a radiolabeled analog of the glycoprotein interleukin-2 (IL2). IL2 is synthesized and secreted in vivo by activated T lymphocytes. IL2 binds with high affinity and specificity to the high affinity cell membrane IL2 receptor, which contains all three of its subunits: α (CD25), β (CD122) and γ (CD132). CD25 and CD122 form the IL2 binding site, whereas CD132 is mainly responsible for signal transduction. The complete IL2 receptor is mainly over-expressed on activated T lymphocytes. Recombinant IL2 is registered as a drug under the name Proleukin®. [^18^F]FB-IL2 displays in vivo behavior similar to Proleukin® and binds similarly to the IL2 receptors that are overexpressed on activated T lymphocytes. [^18^F]FB-IL2 is under investigation as a tracer for the noninvasive assessment of the infiltration of activated T cells in tumors and sites of inflammation and infection, using positron emission tomography (PET).

IL2 receptors have previously been imaged using Single Photon Emission Computed Tomography (SPECT) with ^99m^Tc or ^123^I-labeled IL2 as the tracer. SPECT with radiolabeled IL2 could detect T cell infiltration in patients with melanoma [1], carcinoma [2] and various inflammatory disorders [3], but the technique has several intrinsic disadvantages. ^99m^Tc-labeled IL2 shows high non-specific uptake in liver, spleen, kidneys and bladder [4], which hampers the detection of metastases in or near these organs. SPECT has a low spatial resolution and sensitivity, which hampers detection of small lesions or lesions with low to moderate T cell infiltration. Also, absolute quantification of the imaging signal is difficult. These complications hampered widespread application of SPECT with radiolabeled IL2. To overcome the limitations of SPECT, [^18^F]FB-IL2 was developed as a tracer for PET imaging of IL2 receptors on activated T cells. PET allows absolute quantification of tracer uptake, which is proportional to the number of activated T cells. PET offers several orders of magnitude better sensitivity and approximately 2-fold better spatial resolution than SPECT. The combination of higher sensitivity with a better spatial resolution results in a higher signal-to-noise ratio and more importantly, a higher contrast-to-noise ratio. This alone will result in a higher sensitivity and specificity in clinical studies. Simultaneous acquisition of PET and CT enables accurate localization of the activated T cell signal.

In oncology, [^18^F]FB-IL2 PET can be a useful noninvasive imaging method that can provide information about infiltrating, activated T lymphocytes in all tumor lesions throughout the body, without requiring biopsy. Biopsy methods can only sample a small part of a limited number of tumors. The advantages of in vivo assessment of tumor-infiltrating activated T lymphocytes include avoiding sampling error, assessing the entire tumor volume rather than part of the tumor (addressing the heterogeneity of lymphocyte distribution throughout the tumor), simultaneous assessment of all lesions throughout the body and assessing the immune status of the tumor at diagnosis and in response to treatment. This imaging information may prove useful for patient stratification, selection of patients eligible for immunotherapy, early response prediction and follow-up in patients with various types of cancer. In addition, [^18^F]FB-IL2 PET may allow prediction of adverse side-effects due to lymphocyte activation and infiltration in healthy tissues.

## 2. CHEMICAL PHARMACEUTICAL AND BIOLOGICAL DATA

## 2.1 CHEMICAL PHARMACEUTICAL DATA

### 2.1.S DRUG SUBSTANCE

#### 2.1.S.1 General information

[^18^F]FB-IL2 is a radiolabeled analogue of the cytokine interleukin-2 (Proleukin®), which is a small single-chain glycoprotein (15 kDa) of 133 amino acids. Proleukin® is a registered drug for the treatment of renal cell carcinoma. General information about Proleukin® is described in the SmPC in appendix A (in Dutch). [^18^F]FB-IL2 is under investigation as a tracer for the assessment of the infiltration of activated T cells by noninvasive PET imaging. [^18^F]FB-IL2 is produced as a sterile, intravenously injectable aqueous solution of the radiolabeled protein, containing ca. 100 g/L ethanol, 4.5% glucose, 0.5% human serum albumin (HSA), 0.09% sodium dodecylsulfate (SDS) and 0.02% phosphoric acid. The recommended injected dose of [^18^F]FB-IL2 is 200 MBq, and the mass of injected drug is ≤50 µg (≤3.3 nmol). The medicinal product is no more than a 200 MBq dose of the [^18^F]FB-IL2 solution, diluted with 0.9% sodium chloride to a final volume of approximately 10 mL and filled out in a syringe.

##### 2.1.S.1.1 Nomenclature

*N*-(4-[^18^F]fluorobenzoyl)interleukin-2 for Injection

Abbreviations:

[^18^F]FB-IL2 = *N*-(4-[^18^F]fluorobenzoyl)interleukin-2

[^18^F]FB = 4-[^18^F]fluorobenzoyl

IL2 = interleukin 2 (Proleukin®; aldesleukin)

##### 2.1.S.1.2 Structure

The active substance of Proleukin® (human IL2) is a glycoprotein that is produced by recombinant DNA technology, using an *Escherichia coli* strain which contains the human IL2 gene. [^18^F]FB-IL2 is a radiolabeled analogue of this recombinant human IL2. The radioactive label in [^18^F]FB-IL2 consists of a 4-[^18^F]fluorobenzoyl moiety (C_7_H_4_O^18^F, molecular weight 122 D) that is covalently attached to the primary amino group of a lysine residue of the IL2 protein (molecular weight ca.15,000 D). In [^18^F]FB-IL2, approximately 1.5 moieties of 4-[^18^F]fluorobenzoyl are conjugated on average to each protein molecule [5].

*Figure 1 Structure of [^18^F]FB-IL2 (relative size of the protein is strongly reduced))*

##### 2.1.S.1.3 General Properties

Description: [^18^F]FB-IL2 is a clear and colorless sterile solution of [^18^F]FB-IL2 API in an aqueous solution with a maximum of ca. 100 g/L ethanol, 4.5% glucose, 0.09% SDS, 0.02% phosphoric acid and 0.5% HSA for diagnostic use.

Stereochemistry: Not applicable

Crystal form: Not applicable

Melting range: Not applicable

Hygroscopicity: Not applicable

[^18^F]FB-IL2 consists of Proleukin® radiolabeled with fluorine-18 via a 4-[^18^F]fluorobenzoyl prosthetic group. Fluorine-18 is a radioisotope with 97% positron decay. Positrons have a maximum energy of 0.634 MeV. The decay characteristics of fluorine-18 are favorable for PET imaging, since it has no concomitant decay via emission of gamma photons with an energy near 511 KeV (99.9% - 909 KeV) that could interfere with the coincidence detection of positrons, thus allowing quantitative accuracy of the imaging method. Fluorine-18 has a physical half-life of 109.8 min. It is therefore an attractive positron emitter for labeling of small molecule tracers for PET.

The properties of [^18^F]FB-IL2 are similar to those of Proleukin®, since the radioactive label does not significantly perturb the structure of the protein. The half-life of fluorine-18 (109.8 min) is compatible with the time needed for [^18^F]FB-IL2 to achieve optimal target accumulation and clearance from non-target tissues.

#### 2.1.S.2 Manufacture

##### 2.1.S.2.1 Manufacturer(s)

Proleukin®

In the Netherlands, Proleukin® has been registered since 1989 and approved for treatment of renal cell carcinoma by the Medicine Evaluation Board (“College ter beoordeling van Geneesmiddelen”; RVG 13354). The marketing Authorization Holder of IL2 is Novartis Pharma B.V. IL2 is marketed as Proleukin® 18x10^6^ IE powder for solution for injection or infusion.

Address:

Novartis Pharma B.V.

Raapopseweg 1

6824 DP Arnhem

[^18^F]FB-IL2

[^18^F]FB-IL2 is manufactured from Proleukin® at the department of Nuclear Medicine and Molecular Imaging, University Medical Center Groningen, The Netherlands. Production is performed under the responsibility of the Hospital Pharmacy (manufacturing license nr: 108964F). A map of the production facility can be provided upon request.

Address:

University Medical Center Groningen

Hanzeplein1

9700 RB Groningen

The Netherlands

##### 2.1.S.2.2 Description of Manufacturing Process and Process Controls

[^18^F]fluoride is prepared with a cyclotron by irradiation of [^18^O]water with high-energy protons according to the nuclear reaction: ^18^O(p,n)^18^F. The cyclotron-produced [^18^F]fluoride is allowed to react with ethyl 4-[trimethylammonium]benzoate in DMF yielding ethyl 4-[^18^F]fluorobenzoate. This ester is in situ saponified with sodium hydroxide. After acidification with hydrochloric acid, 4-[^18^F]fluorobenzoic acid is purified by solid phase extraction and allowed to react with O-(N-succinimidyl)-1,1,3,3-tetramethyluronium tetrafluoroborate, yielding N-succinimidyl 4-[^18^F]fluorobenzoate ([^18^F]SFB). The activated ester [^18^F]SFB is purified by high performance liquid chromatography (HPLC), sterilized by filtration and subsequently conjugated to human recombinant IL2 (Proleukin®) in a mixture of ethanol and borate buffer. The product is purified by solid phase extraction and sterilized by filtration over a 0.22 µm filter into a sterile multi-dose vial.

The production of the intermediate [^18^F]SFB conducted in the PET R&D facility of the department of NGMB of the UMCG. The sterilized intermediate is transferred to the GMP unit of the department where it is used as a reagent in the conjugation step. The conjugation, purification and sterilization of the final product is fully automated using an automated synthesis module with disposable cassettes to avoid cross-contamination.

[^18^F]FB-IL2 is produced as a sterile, intravenously injectable aqueous solution with a volume of approximately 10 ml, containing ca. 100 g/L ethanol, 4.5% glucose, 0.5% human serum albumin (HSA), 0.09% sodium dodecylsulfate (SDS) and 0.02% phosphoric acid. Due to the short half-life of the isotope ^18^F (T½ = 110 minutes), a new batch of the radiopharmaceutical has to prepared in-house for each individual patient on the day the PET scan is scheduled. Each batch production generates 1 vial of the drug substance, from which theoretically a maximum of 2 patient doses can be drawn. The recommended dose of [^18^F]FB-IL2 is 200 MBq at the time of injection, and the mass of injected drug substance is ≤50 µg (≤3.3 nmol).

*
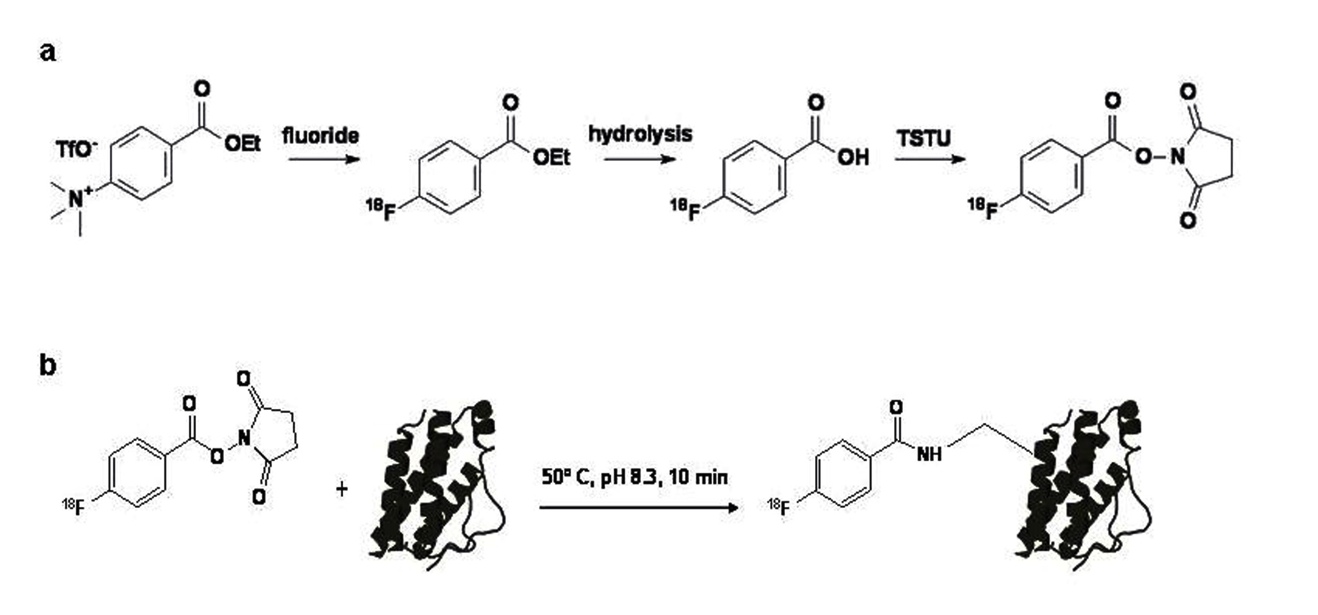
*

*Figure 2 Schematic of the radiosynthesis of [18F]FB-IL2, consisting of 2 steps: a) preparation of the activated ester [18F]SFB and b) conjugation of [18F]SFB to IL2.*

##### 2.1.S.2.3 Control of Materials

The materials used in the preparation of a typical batch via the “[^18^F]SFB conjugation” method, using an Eckert&Ziegler disposable cassette radiosynthesis module, are listed in Table 1.

Recombinant human interleukin-2 is used a precursor for the labeling. The commercially available and approved drug product Proleukin® is used for this purpose. For a summary of product specifications of Proleukin®, see appendix A. Human serum albumin is used for the formulation of [^18^F]FB-IL2. The commercially available and approved drug product Albuman® is used for this purpose. No other raw materials of human or animal origin are used in the radiolabeling process.

All starting materials are checked and released by the responsible hospital pharmacist based on Certificates of Analysis provided with the materials by the supplier or analysis results written on the label on the packaging of the materials.

*Table 1: List of materials used in a [^18^F]FB-IL2 batch production*

| ***Material*** | ***Grade*** |
| --- | --- |
| Proleukin (interleukin-2) | Medicinal product |
| [^18^F]SFB | In-house production >90%, sterile |
| Sterile water | Medicinal product |
| Sodium tetraborate decahydrate | ACS reagent, ≥99.5% |
| Ethanol | ACS, ISO, reagent Ph Eur |
| 0.9% sodium chloride solution | Medicinal product |
| 5% glucose solution (Viaflo) | Medicinal product |
| Albuman, 200 g/l (HSA) | Medicinal product |
| Phosphoric acid, 85% | 85 wt.% in H_2_O, 99.99% trace metals basis |
| Sodium dodecylsulfate | ACS reagent, ≥99.0% |

##### 2.1.S.2.4 Controls of Critical Steps and Intermediates

In process controls for the production of intermediate [^18^F]SFB are listed in table 2. These are all tests performed on the intermediate [^18^F]SFB .

Because of the short half-life and the high radiation field emitted by the ^18^F radioisotope, no in-process sampling points or in-process tests are performed during the subsequent production of [^18^F]FB-IL2, which is a remote controlled, automated process. The conjugation of [^18^F]SFB to IL2 will be performed in a closed, shielded hotcell. The progress of the production can be monitored real-time via the read-out of various radioactivity and temperature detectors in the synthesis module. Read-outs of these detectors are logged in the batch record of the production.

A post-filtration filter integrity test is performed as an in-process control to assure effective filtration prior to release of the drug product.

*Table 2: Requirements for the intermediate [^18^F]SFB*

| **[^18^F]SFB** | **Specification** |
| --- | --- |
| Appearance | Clear, colorless |
| Minimum yield | 5 GB |
| Radiochemical purity | >90% |

#####

##### 2.1.S.2.5 Process validation and/or Evaluation

The process development work which has been carried out has shown that the proposed manufacturing process is suitable and yields consistently a product of the desired quality as laid down in this IMPD. Before the product was administered to humans, four consecutive batches of [^18^F]FB-IL2 were produced and tested according to all quality specifications. See [section 2.1.P. 5.4](#BatchAnalyses21p54) for and overview of results.

##### 2.1.S.2.6 Manufacturing Process Development

The manufacturing processes of [^18^F]FB-IL2 is based on the method that was described by di Gialleonardo et al. (1). The conjugation is implemented in the GMP facilities at the department of NMMI at the UMCG and adopted to establish a fully automated procedure using a synthesis module with a disposable cassette system. Documentation of the process development is included in the product dossier of [^18^F]FB-IL2. The process validation is done at the same time as product validation, see 2.1.S.2.5. This is according to our in-house controlled SOP “Validatie Master Plan 2 (VMP2); Validatie Radiofarmaca”.

#### 2.1.S.3 Characterization

##### 2.1.S.3.1 Elucidation of Structure and Other Characteristics

UPLC

Proleukin® is applied as the reference standard for analysis and the precursor for the labeling. The identity of the [^18^F]FB-IL2 was verified by comparison with an IL2 reference sample using UPLC. The conjugation of 4-[^18^F]fluorobenzoyl moieties to IL2 renders the product slightly more lipophilic, resulting in a small shift in retention time between native IL2 and [^18^F]FB-IL2.


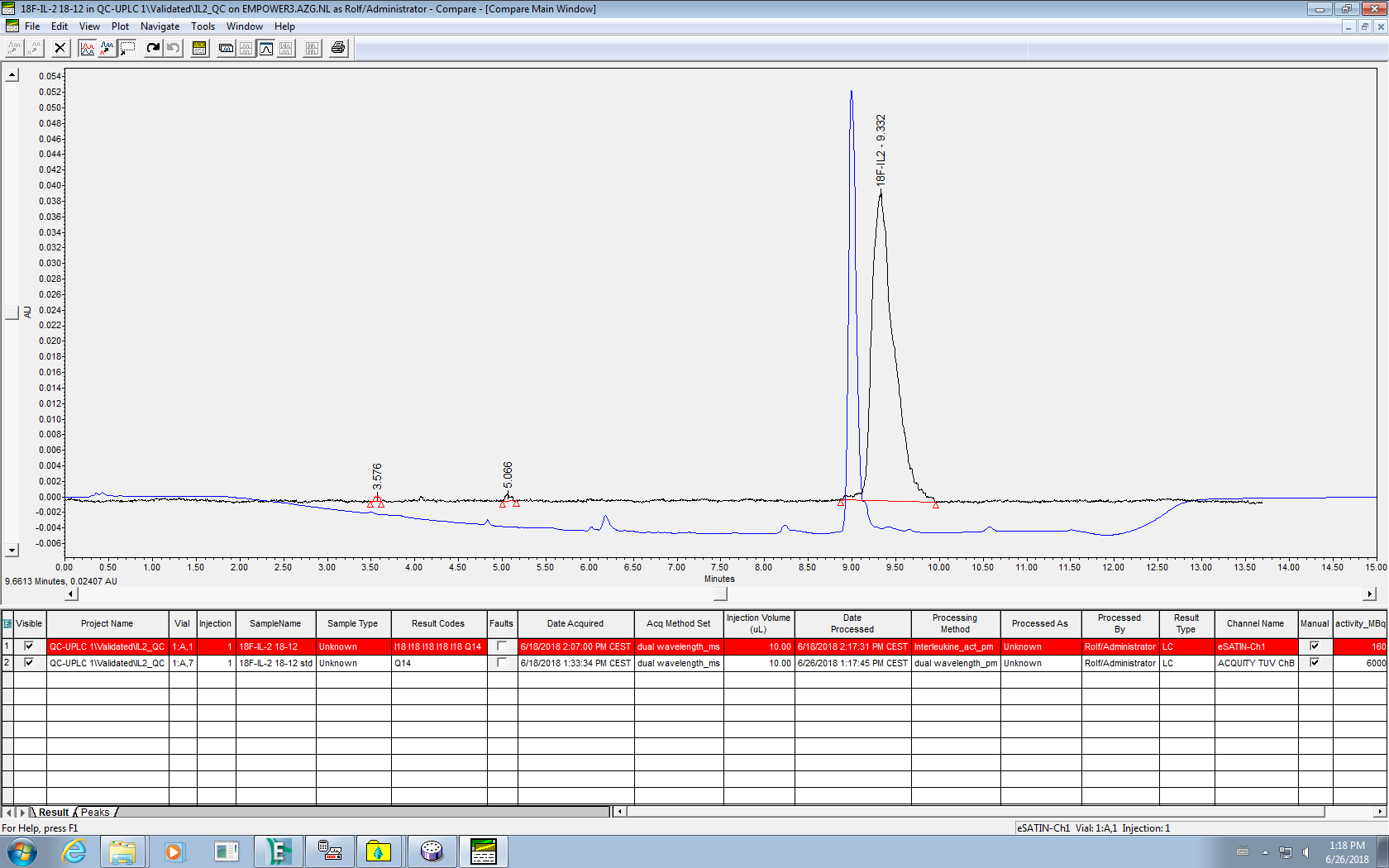


*Figure 3: UPLC chromatogram of Proleukin® (UV signal, blue line) and [^18^F]FB-IL2 (radioactivity signal, black line).*

Mass spectrometry: MALDI-ToF-MS

For identification of labelled IL2, matrix assisted laser desorption/ionization time-of-flight mass spectrometry (MALDI-TOF-MS) was applied on decayed [^18^F]FB-IL2. The mass spectrum showed a single peak corresponding to a molecular weight of approximately 15.5 kD) (Figure 4). This molecular weight is slightly higher than the molecular weight of native IL2 (15.3 kD) due to the conjugation of 1 to 2 4-[^18^F]fluorobenzoyl moieties [5].


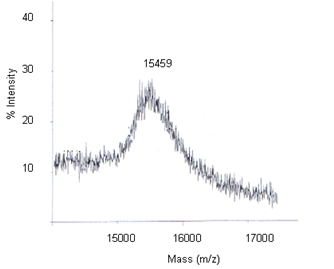


*Figure 4: Characterization of [^18^F]FB-IL2 by MALDI-ToF*

Protein integrity: Gel electrophoresis

Gel electrophoresis separates proteins according to their electrophoretic mobility, which is related to the protein size or molecular weight. Non-reducing conditions allow visualization of aggregates as the result of disulfide bond formation. Gel electrophoresis was performed under non-denaturating conditions by loading 3 µg of native IL2 (lane 1), unpurified reaction mixture of IL2 labeling (lane 2) and purified [^18^F]FB-IL2 (lane 3 and 4) on two 12.5% polyacrylamide gel. Gels were run on a mini gel electrophoresis apparatus. The first gel was stained with Coomassie Brilliant Blue. The second gel was exposed to a phosphor storage screen to detect radioactive proteins by autoradiography. Bands of the Proleukin® and [^18^F]FB-IL2 appeared at the same height on the gel, indicating that both proteins had similar molecular weight. No other radioactive bands were observed, indicating that [^18^F]FB-IL2 remained intact during labeling and does not form irreversible aggregates [5].


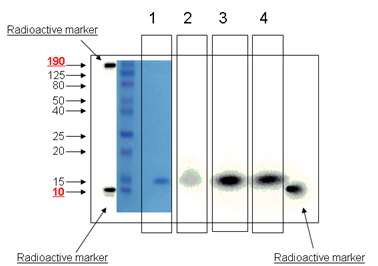


*Figure 5:Gel electrophoresis of Proleukin® (lane 1), unpurified reaction mixture of IL2 labeling (lane 2) and purified [18F]FB-IL2 (lane 3 and 4). The Coomassie Brilliant Blue image is superimposed on the autoradiogram of the gel.*

Biological activity: MTT assay

IL2 stimulates the proliferation of T lymphocytes. In order to test whether the biological activity of [^18^F]FB-IL2 is not compromised by the labeling process, the ability of [^18^F]FB-IL2 to stimulate the proliferation of peripheral blood mononuclear cells (PBMCs) was assessed by an MTT assay. The MTT assay is a colorimetric assay that measures the activity of enzymes that reduce MTT (3-(4,5-dimethulthiazol-2-yl)-2,5-diphenylstetrazolium bromide) into insoluble formazan; it is commonly used to assess cell viability and proliferation. Isolated PBMCs were incubated for 48 hours with 5 µg/ml of phytohemoagglutinin (PHA) to activate them. Cells were incubated for 24 h with different concentrations (0,1; 1; 10; 100; 1000 units/ml) of either native IL2 or decayed [^18^F]FB-IL2. Then, the MTT was performed to compare the cellular proliferation of cells treated with decayed [^18^F]FB-IL2 with that of cells treated with native IL2. Figure 6 shows that native IL-2 and decayed [^18^F]FB-IL-2 display comparable biological activity, as they enhanced proliferation of PHA-activated PBMCs in an equivalent manner [5].


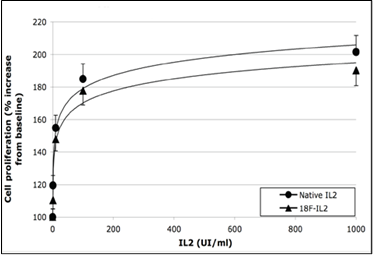


*Figure 6: MTT assay on PHA-activated PBMCs stimulated by either native IL2 or decay [^18^F]FB-IL2.*

##### 2.1.S.3.2 Impurities

Possible known impurities are sodium [^18^F]fluoride, the intermediate 4-[^18^F]SFB and its hydrolysis product 4-[^18^F]fluorobenzoic acid. The total amount of radioactive impurities should be less than 5%. The total amount of unknown nonradioactive impurities should be less than 1 mg/L (assuming these impurities have the same UV absorption coefficient as SFB). As no dedicated Ph. Eur. monograph is available, these acceptance criteria are based on past experience with other radiopharmaceuticals and the generally accepted threshold for unknown impurities in this field. The analysis of radioactive and unknown impurities is performed using UPLC.

Other potential impurities could be residual solvents such as N,N-dimethylformamide and acetonitrile, which are the reaction solvent and the HPLC solvent used in the production of 4-[^18^F]SFB, respectively. The limits for residual solvents are defined in the document “EMEA note for guidance on impurities: residual solvents” (CPMP/ICH/283/95), and in chapter 5.4 of Ph. Eur. The threshold for the presence of acetonitrile and N,N-dimethylformamide are set accordingly at <410 mg/l and <5000 mg/l, respectively. The analysis of residual solvents is performed using gas-chromatography.

#### 2.1.S.4 Control of Drug Substance

Due to the short half-life of ^18^F (110 minutes) and consequently the limited time available to complete the productions, only the final drug product is analyzed. Details on methods for [^18^F]FB-IL2 analysis, their validation, the batch analysis, and the justification of specifications will be provided in the appropriate 2.1.P sub-sections.

##### 2.1.S.4.1 Specification

See section 2.1.P.5.1.

##### 2.1.S.4.2 Analytical Procedures

See section [2.1.P.5.2](#AnalProc21p52).

##### 2.1.S.4.3 Validation of Analytical Procedures.

See section [2.1.P.5.3](#ValAnalProc21p53).

##### 2.1.S.4.4 Batch Analyses

See section [2.1.P.5.4](#BatchAnalyses21p54).

##### 2.1.S.4.5 Justification of specifications for drug substance

See section [2.1.P.5.6](#JustSpec21p56).

#### 2.1.S.5 Reference Standards or Materials

Proleukin® is used as reference standard and produced by Novartis as drug for the treatment of metastatic renal cell carcinoma. The reference standard for the intermediate [^18^F]SFB is purchased from ABX, Germany (catalogue number 4392) and is provided with a certificate of analysis.

#### 2.1.S.6 Container Closure System

After sterile filtration, [^18^F]FB-IL2 is transferred to a glass vial, Ph. Eur. type I, sterile and pyrogen free, covered with a bromobutyl rubber stopper (Ph. Eur. conform), sealed with a flip-off aluminum cap.

Stability tests (see section 2.1.P.7) do not show any indication of incompatibilities with the primary packaging with the drug substance.

### 2.1.S.7 Stability

The drug substance is prepared and formulated into the drug product as a continuous uninterrupted process. Stability testing of this material is performed on the finished drug product solution. See section 2.1.P.7.

During development, the in-vitro stability of [^18^F]FB-IL2 was tested in phosphate buffered saline (PBS) and in human plasma using the trichloroacetic acid (TCA) precipitation test. Purified [^18^F]FB-IL2 was dissolved in 5 ml of human plasma or PBS and samples were taken repetitively. The proteins in these samples were precipitated with 20% of TCA and centrifuged. Low molecular weight ^18^F-labeled fragments appear in the supernatant and the protein bound radioactivity is present in the pellet. As shown in the figure 7, 95±3% of the labeled product is still intact for at least 2 hours of incubation in PBS. In human plasma, no degradation was observed at all. Thus, the stability of the tracer appears sufficient for imaging purposes [5]. The label on the dispensing vial will include an expiration time of 60 minutes.


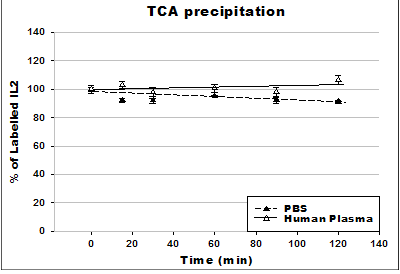


*Figure 7: TCA Precipitation test of [^18^F]FB-IL-2 in PBS and human plasma*

### 2.1.P MEDICINAL PRODUCT

#### 2.1.P.1 Description and Composition of the DRUG Product

Please refer to section 2.1.S, as the medicinal product is no more than the recommended dose of 200 MBq of the produced drug substance (consisting of the radiopharmaceutical, dissolved in ca. 100 g/L ethanol, 4.5% glucose, 0.09% SDS, 0.02% phosphoric acid and 0.5% HSA) at the time of injection, diluted with 0.9% sodium chloride to a volume of approximately 10 ml.

*Table 3: Composition of a Batch of Drug Product [^18F^]FB-IL2 (approximately 10 ml)*

| **Name of ingredient** | **Composition** | **Function** | **Reference to standard** |
| --- | --- | --- | --- |
| Active ingredients: |  |  |  |
| [^18^F]FB-IL2 | 100-300 MBq | Active ingredient | In house production. Concentration depends on cyclotron irradiation time, radiochemical yield and radioactive decay |
| Other ingredients: ^a^ |  |  |  |
| - Sodium chloride 0.9% - Ethanol - Glucose 5% - Sodium dodecylsulfate | 0 – 9 mL  0.1 – 0.9 mL  1 – 11 mL  1 – 10 mg | Diluent  Solvent  Solvent  Exipient | Ph.Eur.  Ph.Eur.  Ph.Eur. |
| - Human serum albumin 200 g/L - Phosphoric acid | 0.03 – 0.30 mL  0.001 – 0.012 µL | Exipient  Exipient |  |
|  |  |  |  |

^a^ The exact composition of the drug product depends on the dilution factor of the drug substance with 0.9% NaCl solution that is required to obtain the recommended radioactivity dose (200 MBq) in an injection volume of 8 - 13 ml. The ranges given in the table represent the situation where no dilution of the drug substance is required and the situation where the drug substance has to be diluted 10-fold with saline.

#### 2.1.P.2 Pharmaceutical development

##### 2.1.P.2.1 Components of the Medicinal Product

The medicinal product consists of a solution of 100 – 300 MBq [^18^F]FB-IL2 in approximately 100 g/L ethanol, 4.5% glucose, 0.09% SDS, 0.02% phosphoric acid and 0.5% HSA, diluted with 0.9% sodium chloride to a total volume of ca. 10 mL.

##### 2.1.P.2.2 Medicinal product

The [^18^F]FB-IL2 formulation is a sterile, intravenous injectable aqueous solution to be used for detection and visualization of activated T lymphocytes with PET.

##### 2.1.P.2.3 Manufacturing process development

The appropriate dose of the drug substance (200 MBq) is dispensed in a syringe and diluted with 0.9% NaCl to the injection volume of ca. 10 mL. The radioactive dose of the active ingredient is measured with a dose calibrator and corrected for radioactive decay between the measurement and administration to the patient. Administration of the drug product will be done in-house by a qualified professional immediately after production (i.e. within the expiration time of 60 minutes). Documentation of the process development is included in the product dossier of [^18^F]FB-IL2.

##### 2.1.P.2.4 Container Closure System

The drug is dispensed in a 10 mL syringe and transported in a lead shielded holder.

##### 2.1.P.2.5 Microbiological Attributes

The drug substance is sterilized by filtration over a 0.22 µm filter under aseptic conditions. The drug product is prepared by dilution of the drug substance in a laminar airflow cabinet. The drug product is sterile and free of endotoxins, as demonstrated by analysis of validation batches.

##### 2.1.P.2.6 Compatibility

[^18^F]FB-IL2 is a lipophilic protein that can form tetramers, especially under neutral to basic conditions. Aggregation of the tracer is reversible, but can be avoided when a formulation is used that contains ethanol glucose, SDS and HSA. The absence of aggregates in the formulation was confirmed by gel electrophoresis (see 2.1.S.3.1) and by the UPLC. After injection of the drug product on a UPLC column, the aggregates would remain trapped on the column. However, the recovery of radioactivity from the UPLC column was 95% (as determined during validation of the QC method), indicating that the amount of aggregates trapped on the column is <5%. At this moment, no cross reactions with other drug substances have been described. When the drug product is administered via a catheter, some retention of the drug substance in the catheter may occur. Lines should be as short as possible. Retention of drug substance in the syringe and lines is measured after the administration in order to calculate the net administered dose, as is standard procedure for the administration of injectable radiopharmaceuticals in clinical trials.

#### 2.1.P.3 Manufacture

##### 2.1.P.3.1 Manufacturer

See 2.1.S.2.1

##### 2.1.P.3.2 Batch Formula

A batch of [^18^F]FB-IL2 is represented by a single, multi-dose vial with an expiration time of 60 minutes. Batches are freshly prepared on the day of administration. The exact composition of the drug product depends on the dilution factor of the drug substance with 0.9% NaCl solution that is required to obtain the recommended radioactivity dose (200 MBq) in an injection volume of ca. 10 mL. The ranges given in table 4 represent the situation where no dilution of the drug substance is required and the situation where the drug substance has to be diluted 10-fold with saline.

*Table 4: Batch formula of [^18^F]FB-IL2*

| **Name of ingredient** | **Composition** |
| --- | --- |
| Active ingredients: |  |
| [^18^F]FB-IL2 | 100-300 MBq |
| Other ingredients: |  |
|  |  |
| - Sodium chloride 0.9% - Ethanol - Glucose 5% - Sodium dodecylsulfate | 0 – 9 mL  0.1 – 0.9 mL  1 – 11 mL  1 – 10 mg |
| - Human serum albumin 200 g/L - Phosphoric acid 85% | 0.03 – 0.30 mL  0.001 – 0.012 µL |

##### 2.1.P.3.3 Description of manufacturing process

See 2.1.S.2.2. for manufacturing of the drug substance.

The formulated drug substance solution, consisting of [^18^F]FB-IL2 in approximately 100 g/L ethanol, 4.5% glucose, 0.09% SDS, 0.02% phosphoric acid and 0.5% HSA, is sterilized by filtration over a 0.22 μm filter and collected in a sterile vial. The radioactivity concentration in the sterile vial with the drug substance solution is measured with a dose calibrator. A sample of this solution is taken for quality control measurements, as described in section 2.1.P.5.1. The drug product is prepared by aspiration of the required volume of the drug substance solution into a syringe in a laminar flow cabinet. The required volume of the drug substance solution is calculation based on the radioactivity concentration of the drug substance in the sterile vial, corrected for radioactive decay, and the time interval between measurement and the administration of the drug product to the patient. The recommended dose at the time of administration is 200 MBq. The drug substance in the syringe is subsequently diluted by aspiration of 0.9% NaCl solution to obtain a total injection volume of ca. 10 mL, thus obtaining the final drug product. The amount of radioactivity in the syringe is measured in a dose calibrator, which is linked to the laboratory management system IBC that produces the label with drug product and patient information for this specific dose. The syringe is placed in a lead shielded holder to avoid exposure to radioactivity by the staff. The drug product is intravenously injected to the patient by a qualified professional within 1 hour after production of the drug substance.

#####

##### 2.1.P.3.4 Control of critical steps and intermediates

Due to the short half-life of [^18^F]FB-IL2, no process controls of intermediates can be performed. Instead, quality control is performed on the drug product itself for every batch production.

##### 2.1.P.3.5 Process Validation and/ or Evaluation

Control of the drug substance and drug product are performed simultaneously, since the drug product is identical to the drug substance, diluted in 0.9% NaCl (See section 2.1.S.2.5). This is according to our local procedure ‘Validatie Master Plan 2 (VMP2); Validatie Radiofarmaca’.

#### 2.1.P.4 Control of Excipients

##### 2.1.P.4.1 Specifications

Excipients are listed in Table 5.

*Table 5: List of excipients in the [^18^F]FB-IL2 drug product solution*

| **Excipient** | **Supplier (article number)** | **Grade** | **Amount** |
| --- | --- | --- | --- |
| Ethanol | Merck (art. 1.00983) | ACS, ISO, reagent Ph Eur | 0.1 – 0.9 mL |
| Glucose, 5% (Viaflo) | Baxter BV | Medicinal product | 1 – 11 mL |
| Sodium dodecylsulfate | Sigma Aldrich (art. 436143) | ACS reagent, ≥99.0% | 1 – 10 mg |
| Albuman, 200 g/L | Sanquin Plasma Products BV | Medicinal product | 0.03 – 0.30 mL |
| Phosphoric acid | Sigma Aldrich (art.345245) | ACS reagent, ≥85% | 0.001 – 0.012 µL |
| NaCl, 0.9% | B. Braun | Medicinal product | 0 – 9 mL |

##### 2.1.P.4.2 Analytical procedures

The excipients are approved by the QP, following a check for packaging integrity, expiry and Certificate of Analysis. The appearance should also be verified. For these reasons, there is no need to describe analytical procedures for excipient control.

##### 2.1.P.4.3 Validation of Analytical Procedures

Not applicable.

##### 2.1.P.4.4 Justification of Specifications

Not applicable.

##### 2.1.P.4.5 Excipients of Human or Animal Origin

Human serum albumin is applied as exipient to prevent the aggregation of [^18^F]FB-IL2. Human serum albumin is registered as a medicinal product in the Netherlands (trade name Albuman, registration number RVG 103594).

#####

##### 2.1.P.4.6 Novel Excipients

Not applicable.

####

#### 2.1.P.5 Control of DRUG product

##### 2.1.P.5.1 Specifications

*Table 6: Specifications of [^18^F]FB-IL2*

| **Test** | **Method** | **Acceptance criteria** | **Absolute amount^1^** |
| --- | --- | --- | --- |
| Appearance | Visual inspection | Clear, colorless |  |
| pH | pH indicator paper | 4 – 7 |  |
| Radiochemical Identity^2^ | UPLC | Retention time of Proleukin reference standard + (0.6–1.0 min) |  |
| Radiochemical Purity | UPLC | ≥ 95% |  |
| Radionuclide Identity | Germanium detector | Half-life=105 – 115 min  Energy=511 keV |  |
| Radionuclide Purity | Germanium detector | ≥ 99% |  |
| Molar Activity | UPLC | ≥ 50,000 MBq/µmol |  |
| [^18^F]FB-IL2 Concentration^3^ | UPLC | < 5 mg/L | < 50 µg |
| Unknown Impurities | UPLC | < 1 mg/L | < 10 µg |
| Kryptofix | TLC spot test | < 25 mg/L | < 250 µg |
| Acetonitrile | GC | < 410 mg/L | < 4 mg |
| DMF | GC | <880 mg/L | < 8.8 mg |
| Ethanol | GC | < 150 g/L | <1.5 g |
| Osmolarity | Osmometer | < 3,000 (mosmol/kg) |  |
| Bacterial Endotoxins^4^ | LAL test | < 2.5 EU/mL | < 25 EU |
| Sterility | Bacterial growth in broth | Sterile |  |
| Filter integrity^5^ | Bubble point test | > 3.4 bar |  |
|  | Pressure hold test | > 1.8 bar, <10% reduction in pressure |  |

^1^The maximum amount in a typical injection volume of 10 mL is provided, but the actual amounts will be lower since the recommended dose of the drug substance has to be diluted with 0.9% NaCl to a total volume of 10 mL. The dilution factor is variable, since it depends on e.g. the production yield of the drug substance, radioactive decay and the interval between measurement and administration to the patient.

^2^The product [^18^F]FB-IL2 is slightly more lipophilic than Proleukin, hence the retention time is slightly increased

^3^This corresponds to a maximum dose of 50 µg for a standard injection volume of 10 mL.

^4^Ph.Eur. 2.6.14 bacterial endotoxins: 2.5 EU* bodyweight (kg) / Volume. Assuming a worst case scenario of mL and an adult patient weighing 50 kg, this will be 12.5 EU/mL.

^5^Two tests can be performed to check the integrity of the sterilization filter: 1) the pressure hold test before release of the product and 2) the bubble point test after decay of radioactivity. The bubble point test is considered to be the golden standard, but is not performed before release of the product, because of the radiation burden to the operator.

#####

##### 2.1.P.5.2 Analytical Procedures

The panel of analytical tests includes: visual inspection, pH measurement, (radio)chemical purity analysis, radionuclide purity, residual solvent analysis, filter integrity test, molar activity, bacterial endotoxin content, osmolarity and sterility. The analytical procedures are validated and adequate to detect significant deviations from the specifications. In short:

*Appearance*

Visual inspection is performed after final sterile filtration to confirm that the solution is clear and colorless.

*pH*

The pH of the drug product is determined with a pH indicator paper

*Kryptofix*

Kryptofix is determined by the kryptofix spot test using silica TLC strips treated with an aquous iodoplatinate solution. Discoloration of the strip will be compared with a 25 mg/mL kryptofix reference sample.

*(Radio)chemical purity and molar activity*

(Radio)chemical purity and molar activity are determined by UPLC (appendix C). The UPLC system consists of the following components and parameters:

- Pomp: Waters Acquity QSM manager

- Injector: Waters Acquity FTN Sample manager

- UV Detector: Waters Acquity TUV detector

- RA-Detector: Flowstar LB513 met MX 50-6 Cell

- Empower Software Version 3

- Column 4 (ACQUITY UPLC® BEH Shield RP18 1.7μm; (3.0mm x 50mm)

- Mobile phase: gradient elution 5% acetonitrile (+0.1% trifluoroacetic acid) in water 🡪 70% acetonitrile (+0.1% trifluoroacetic acid) in water

- flow:

- UV detection: 225 + 280 nm

- Temperature: 40 ^o^C

- run time: 15 min

10 μL of the QC sample is injected on UPLC for analysis. [^18^F]FB-IL2 will elute from the column at 9-10 min.

Molar activity is calculated from the total radioactivity in the drug product and from the amount of carrier (i.e. non labelled product) as assessed by analytical UV-UPLC with calibration curves.

*Filter Integrity Testing*

The filter integrity test is performed according to pressure hold-test as part of the manufacturing process. For radiation safety reasons, the bubble-point method, according to Eur. Pharmacopeia, will be performed only if the pressure hold test fails.

*Radionuclide purity*

Radionuclide purity will be determined with a germanium detector. Radionuclide identity will be determined for the gamma spectrum emitted by the drug product. Fluorine-18 should only show peaks at 511 and 1022 keV. The half-life will be determined by measuring the radioactive decay over time. The presence of long-lived radioisoptopes will be determined by measuring the sample again 24 hours after production when all fluorine-18 derived radioactivity should have been decayed.

*Residual solvent analysis*

Concentrations of residual solvents are quantified by FID-gas chromatography by use of internal standard and pre-run calibration curves.

*Osmolarity*

The test is performed on the decayed sample using an osmometer.

*Endotoxin content*

Endotoxin levels are assessed by use of a LAL test system according to the instructions provided by the supplier (BioTek Elx808, Cambrex).

*Sterility*

A sterility test is performed by adding a sample of the decayed drug product to TBS medium (Soya-bean casein digest). After 14 days at 25 ^o^C, the clarity of the medium is visually inspected. In case the medium is not clear the sample is sent to the deparment of medical microbiology for determination of the bacterial strain.

##### 2.1.P.5.3 Validation of Analytical Procedures

The analytical procedures have been validated to demonstrate the suitability for use in Drug product testing for clinical supply, according to the internally controlled SOP “Farmaceutische Controle: Validatie; Radiochemische-analysemethoden”. The validation of the analytical procedures for [^18^F]FB-IL2 has been described in Performance Qualification report no. 16-05.

##### 2.1.P.5.4 Batch Analyses

The results of the validation batch analyses are summarized in table 7.

***Table 7: Batch analysis of [^18^F]FB-IL2***

| **Test** | **Acceptance criteria** | **Validation 1**  **31-08-2016** | **Validation 2**  **02-09-2016** | **Validation 3**  **06-09-2016** | **Validation 4**  **09-09-2016** |
| --- | --- | --- | --- | --- | --- |
| Appearance | Clear, colorless | Clear, colorless | Clear, colorless | Clear, colorless | Clear, colorless |
| pH | 4 – 7 | 4.5 | 5 | 4.5 | 7 |
| Radiochemical Identity^1^  - retention time Proleukin  - retention time [^18^F]FB-IL2 | - ca. 9 min  - retention time Proleukin + (0.6–1.0 min) | - 8.9 min  - 9.6 min | - 9.0 min  - 9.7 min | - 8.9 min  - 9.8 min | - 9.0 min  - 9.5 min |
| Radiochemical Purity | ≥ 95% | 97.1% | 97.4% | 95.7% | 98.0% |
| Radionuclide Identity | Half-life=105 – 115 min  Energy=511 keV | 115 min  511 keV | 115 min  511 keV | 112 min  511 keV | 112 min  511 keV |
| Radionuclide Purity | ≥ 99% | ≥ 99% | ≥ 99% | ≥ 99% | ≥ 99% |
| Molar Activity | ≥ 50,000 MBq/µmol | ≥ 50,000 MBq/µmol | ≥ 50,000 MBq/µmol | ≥ 50,000 MBq/µmol | ≥ 50,000 MBq/µmol |
| [^18^F]FB-IL2 Concentration | < 5 mg/L | < 5 mg/L | < 5 mg/L | < 5 mg/L | < 5 mg/L |
| Unknown Impurities | < 1 mg/L | < 1 mg/L | < 1 mg/L | < 1 mg/L | < 1 mg/L |
| Kryptofix | < 25 mg/L | < 25 mg/L | < 25 mg/L | < 25 mg/L | < 25 mg/L |
| Acetonitrile | < 410 mg/L | < 10 mg/L | < 10 mg/L | < 10 mg/L | < 10 mg/L |
| DMF | <880 mg/L | <200 mg/L | <200 mg/L | <200 mg/L | <200 mg/L |
| Ethanol | < 150 g/L | 87.7 g/L | 102.7 g/L | 88.0 g/L | 105 g/L |
| Osmolarity | < 3,000 (mosmol/kg) | 2760 mosmol/kg | 2940 mosmol/kg | 2580 mosmol/kg | 2790 mosmol/kg |
| Bacterial Endotoxins | < 2.5 EU/mL | <0.5 EU/mL | <0.5 EU/mL | <0.5 EU/mL | <0.5 EU/mL |
| Sterility | Sterile | Sterile | Sterile | Sterile | Sterile |
| Filter integrity  - Bubble point | > 3.4 bar | > 3.4 bar | > 3.4 bar | > 3.4 bar | > 3.4 bar |

##### ^1^ Before each analysis the retention time of a Proleukin reference standard is determined. The retention time of [^18^F]FB-IL2 should be 0.6-1.0 minutes longer than that of the Proleukin reference standard, as the PET tracer is slightly more lipophilic that Proleukin.

##### 2.1.P.5.5 Characterization of impurities

Acceptance criteria and analysis methods for expected impurities are listed in in table 8.

*Table 8: Acceptance criteria and analysis methods for expected impurities*

| **Impurity** | **Requirement** | **Analysis method** |
| --- | --- | --- |
| Kryptofix222 | <25 mg/L | TLC spot test |
| [^18^F]fluoride^1^ | < 5% | TLC |
| [^18^F]SFB^1^ | < 5% | UPLC |
| 4-[^18^F]fluorobenzoic acid^1^ | < 5% | UPLC |
| Total unknown impurities | < 1 mg/L | UPLC |
| Acetonitrile | < 410 mg/L | GC |
| DMF | < 880 mg/L | GC |

^1^ Additional requirement: the total amount of radioactive impurities should not exceed 5% (i.e. the radiochemical purity of the product should be ≥95%)

##### 2.1.P.5.6 Justification of Specifications

Specifications were based on the Ph. Eur., literature data and historic data at the manufacturer. See all specifications and their justifications listed in the table 9 below***.***

*Table 9: Justification of specifications of [^18^F]FB-IL2*

| **Parameter** | **Specification** | **Rationale** |
| --- | --- | --- |
| *Pre-Release Test Requirements* | | |
| Appearance | Clear, colorless solution | This is a general requirement applicable to injectable products |
| pH | 4 to 7 | The prescribed pH range is within range of a number of small volume injectables as listed in Ph.Eur. monographs. |
| Radiochemical Purity | ≥ 95% | This is a more stringent requirement than observed in a number of radio-pharmaceutical monographs. |
| Known radiochemical impurities ([^18^F]fluoride, [^18^F]SFB, 4-[^18^F]fluorobenzoic acid) | < 5% | To comply with the specification for the Radiochemical Purity, the amount of each individual radioactive impurity and the sum of all radioactive impurities together should be less than 5% |
| [^18^F]FB-IL2 concentration | < 5 mg/L | This concentration corresponds to a maximum dose of 50 µg (3 nmol) for a standard injection volume of 10 ml. This dose has not given any adverse effect in studies using radiolabeled IL2 for SPECT imaging. |
| Unknown nonradioactive impurities | < 1 mg/L | As no dedicated Ph. Eur. monograph is available, these acceptance criteria are based on past experience with other radiopharmaceuticals and the generally accepted threshold for unknown impurities in this field. |
| Radiochemical Identity | Retention time of [^18^F]FB-IL2 corresponds with the reference standard Proleukin corrected for 0.8 min delay in retention due to increased lipophilicity after labeling. Range: ± 0.2 min | This is a standard requirement to confirm identity by UPLC |
| Radionuclide Identity | T_1/2_ 110 min; allowable range 105 – 115 minutes | This limit is based on Ph. Eur. USP general chapter <823> for compounding 18F containing PET radiopharmaceuticals describes the same requirement for this specification. |
| [^18^F]FB-IL2 Molar Activity | ≥ 50,000 MBq/µmol | This prescribed limit corresponds with a [^18^F]FB-IL2 mass dose of 50 µg at an injected dose of 200 MBq. |
| Kryptofix 222 | ≤ 25 mg/L | The Ph. Eur. asks <25 mg/L as requirement for FDG. The USP requirement is less strict, but the limit complies as well with USP. |
| Endotoxin | ≤ 2.5 EU/mL | Conforms with the 175 EU/V limit specified in USP radio-pharmaceutical monographs.  The Ph. Eur gives a norm, based on infusion fluid/ kg. |
| Filter Integrity (pressure hold) | >1.8 bar on filter. Less than 10% pressure reduction in 30 seconds. | Prerelease test to detect overt defect in the sterilization filter. Test is integrated in the synthesis program and prevents exposure of the operator to radioactivity. |
| **Parameter** | **Specification** | **Rationale [examples]** |
| *Post-Release Test Requirements* | | |
| Filter Integrity (bubble point) | ≥ 3.4.Bar for post-release bubble point test | Conforms to filter manufacturer’s recommendations |
| Solvents | Acetonitrile < 410 mg/mL  DMF < 880 mg/mL  Ethanol < 150 mg/mL | DMF and acetonitrile are conform the Ph. Eur requirement (5.4). The ethanol requirement is based on the anticipated formulation in ca. 100 g/L ethanol. |
| Sterility | Meets current Ph. EU SterilityTest Requirements , paragraph 2.6.1 | Ph. EU requirement for injectables (2.6.1) |
| Osmolarity | <3,000 mOsmol/kg | Based on the formulation consisting mainly of about 100 g/L ethanol, 5% glucose and 0.9% NaCl. Only a small volume will be injected (ca. 10 ml) at a slow infusion rate (Ph. Eur. 2.2.35). |
| Radionuclidic Purity  (Performed on decayed sample) | ≥ 99% | This requirement is slightly more liberal than Ph. Eur and USP monograph for Fludeoxyglucose F18 Injection (>99.9% and >99.5, respectively). The Eur monography for [^18^F]FDOPA states that the result should not be above background. Given the accuracy of the measurement and the fact that radionuclides with half-lifes shorter than 24 h cannot be (accurately) measured, the acceptance criteria was set at ≥ 99% |

##### 2.1.P.5.7 Reference standards

Proleukin® is used as reference standard and produced by Novartis as drug for the treatment of metastatic renal cell carcinoma.

#### 2.1.P.6 Container Closure System

[^18^F]FB-IL2 is contained in a sterile glass vial with septum. These vials consist of type 1 glass and have a bromobutyl stopper. The vial is stored in a lead container to shield the radiation. The required amount of radioactivity (200 MBq [^18^F]FB-IL2) is diluted with 0.9% NaCl up to a total volume of 10 ml. The drug product is filled out in a 10 ml, sterile syringe, consisting of polypropylene (barrel and plunger) and synthetic rubber (plunger stopper, latex free).

#### 2.1.P.7 Stability

Radioactive half-life of [^18^F]FB-IL2 is 110 min. The tracer will be prepared for in-house use only and injection takes place as soon as possible after production. The stability of the product has been investigated on the validation batches using UPLC analysis. The product was stable for at least 1 hour after production in the current formulation. In this period, the radiochemical purity did not decrease more than 0.3%, which is within the test-retest variability of the analysis method (See table 10). The expiration time on the label is therefore set at 1 hour.

*Table 10: Stability data* of [18F]FB-IL2

|  | **Radiochemical purity (%)** | |
| --- | --- | --- |
| **Batch** | **T = 0** | **T = 1 hour** |
| Validation 1  31-08-2016 | 97.1 % | 97.4% |
| Validation 2  02-09-2016 | 97.4% | 97.1% |
| Validation 3  06-09-2016 | 95.7% | 96.2% |
| Validation 4  09-09-2016 | 98.0% | 99.0% |

## 2.2 Non-clinical pharmacology, pharmacokinetics and toxicology

### 2.2.1 Non-clinical pharmacology

In vitro tests

In vitro studies with [^18^F]FB-IL2 were performed at the department of Nuclear Medicine and Molecular Imaging, University Medical Center Groningen, to evaluate possible alterations in the intrinsic properties of the IL-2 molecule after labeling with fluorine-18, including protein stability and binding characteristics [5]. Stability tests included UPLC analysis (see section 2.1.P.7), mass spectrometry, non-denaturing gel electrophoresis (See: section 2.1.S.3.1) and TCA precipitation tests (See: section 2.1.S.7). All tests confirmed the stability of [^18^F]FB-IL2. In vitro investigations of the biological activity of [^18^F]FB-IL2 was based on the MTT test (See: section 2.1.S.3.1). The MTT test demonstrated that the biological activity of [^18^F]FB-IL2 was maintained after labeling.

Ex-vivo biodistribution (Balb/c mice)

The biodistribution of [^18^F]FB-IL2 in Balb/c mice showed low uptake in all organs (SUV<1), with negligible uptake in spleen, stomach and liver (Figure 8). The kidneys were the organs with highest radiotracer uptake and the majority of radioactivity was rapidly excreted in the urine, indicating that the tracer is mainly cleared via the renal pathway, a characteristic that is similar to the native IL2. [^18^F]-FB-IL2 showed low bone uptake, which does not significantly increase over time, indicating that defluorination in-vivo is negligible.


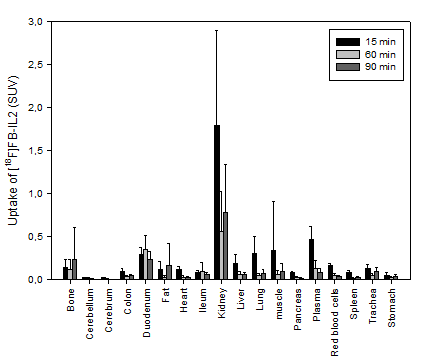


*Figure 8:Ex-vivo biodistribution of [^18^F]FB-IL2 in healthy immune-competent BALB/c mice*

Targeting and Blocking studies (SCID mice)

The severe combined immunodeficiency (SCID) mouse model is characterized by impaired adaptive immune responses due to atypical or absent T and B lymphocytes as result of a genetic disorder. Hence, SCID mice represent a useful model for research into the basic biology of the immune system in response to infection and disease. SCID mice were used to assess [^18^F]FB-IL2 specific targeting of xenografts of PHA-activated human peripheral blood mononuclear cells (PBMCs) [5].

SCID mice were inoculated with increasing amounts of PHA-activated human PMBCs in matrigel in one shoulder and PBS in matrigel as control in the other shoulder. Matrigel is a gelatinous protein that resembles the complex extracellular environment found in many tissues. PET imaging with [^18^F]FB-IL2 demonstrated that the administered PBMCs quickly migrated from the injection site to the contra-lateral shoulder, due to local inflammation induced by injection of matrigel (Figure 9). Migration was confirmed by hematoxylin and eosin (HE) staining of these tissues. A low correlation was found between [^18^F]FB-IL2 uptake and number of cells at the site of PBMC injection (r^2^ =0.09). However, correlation improved dramatically when uptake at both injection and migration sites was combined (r^2^ = 0.75). Blocking studies were performed in SCID mice with xenografts of PHA-activated human PBMCs by pretreatment with a 100-fold excess of native IL2 30 minutes before administration of [^18^F]FB-IL2. Saturation of the IL2 receptor with unlabeled Proleukin® decreased tracer uptake in the xenograft with 72%, demonstrating that tracer uptake is specifically mediated by the IL2 receptor.


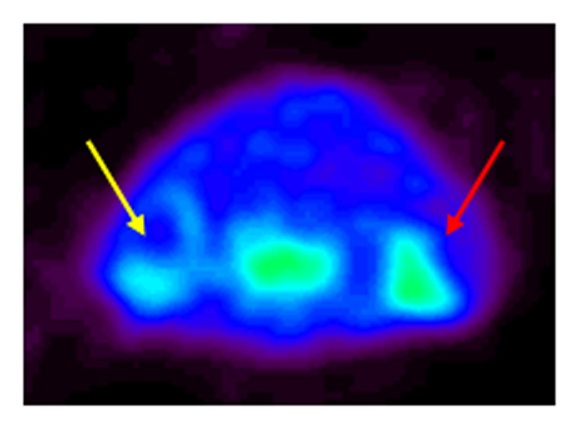

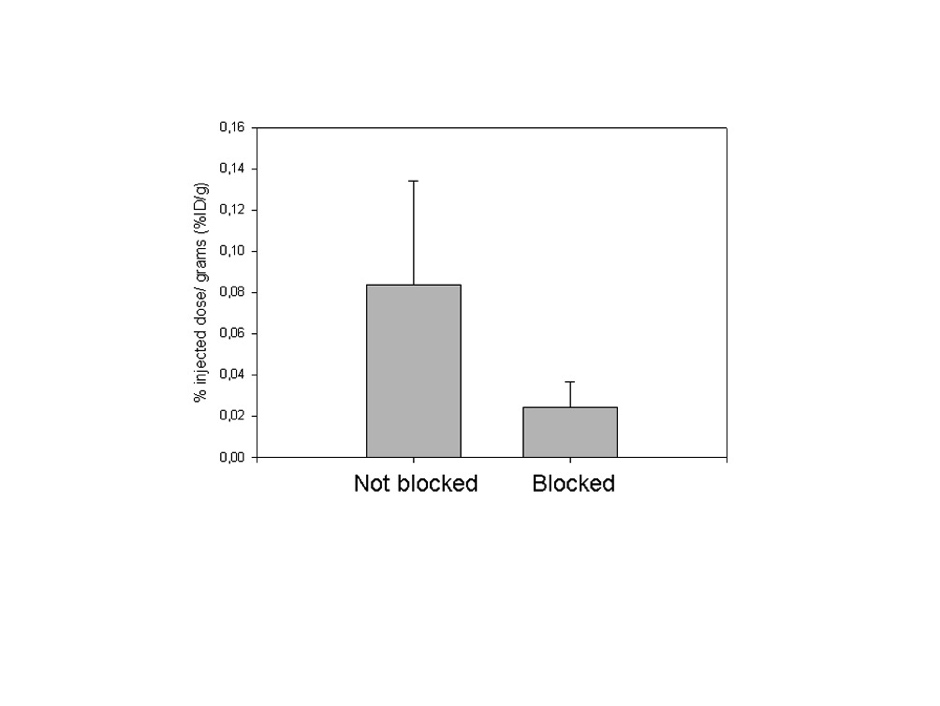


*Figure 9:A) Transaxial PET image, showing the tracer uptake at the injection site of human PBMCs in SCID mice (red arrow) and the migration of PBMC to the contra-lateral side (yellow arrow), due to a local inflammation. B) The effect of pretreatment with a 100-fold excess Proleukin® (Blocked) on the uptake of [^18^F]FB-IL2 in the PBMC xenograft.*

Monitoring of insulitis

Type I diabetes mellitus is an autoimmune disease characterized by chronic hyperglycemia resulting from the destruction of insulin-producing pancreatic beta cells. During the pre-diabetic phase, inflammation develops as a result of pancreatic infiltration of lymphocytes that target and destroy the beta cells. This process is called insulitis and was studied in animal models e.g. non-obese diabetic (NOD) mouse or diabetic prone BB/W rats using [^18^F]FB-IL2 [7]. NOD mice showed increasing pancreatic uptake of [^18^F]FB-IL2 with age and blood glucose levels, thus reflecting the progression of insulitis. Moreover, NOD mice with blood glucose concentrations higher than 8 mmol/l (indicative of significant autoimmune destruction of beta cells) have a 4.3-fold higher radiopharmaceutical uptake in the pancreas compared to NOD mice with a normal blood glucose level (p = 0.0005). [^18^F]FB-IL2 PET imaging could also clearly visualize the pancreas in BB/W rats, although a wide range in pancreatic tracer uptake was observed. Histological examination confirmed that the variable [^18^F]FB-IL2 uptake reflects the variability in the degree of lymphocytes infiltration in the pancreas of the BB/W rats in this study. A strong correlation between the extent of insulitis and the radiopharmaceutical uptake in the pancreas was found (r^2^=0.801).


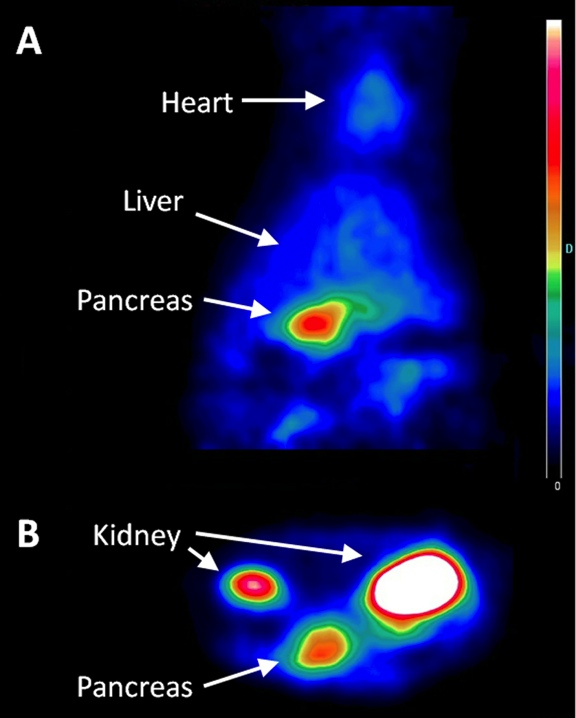

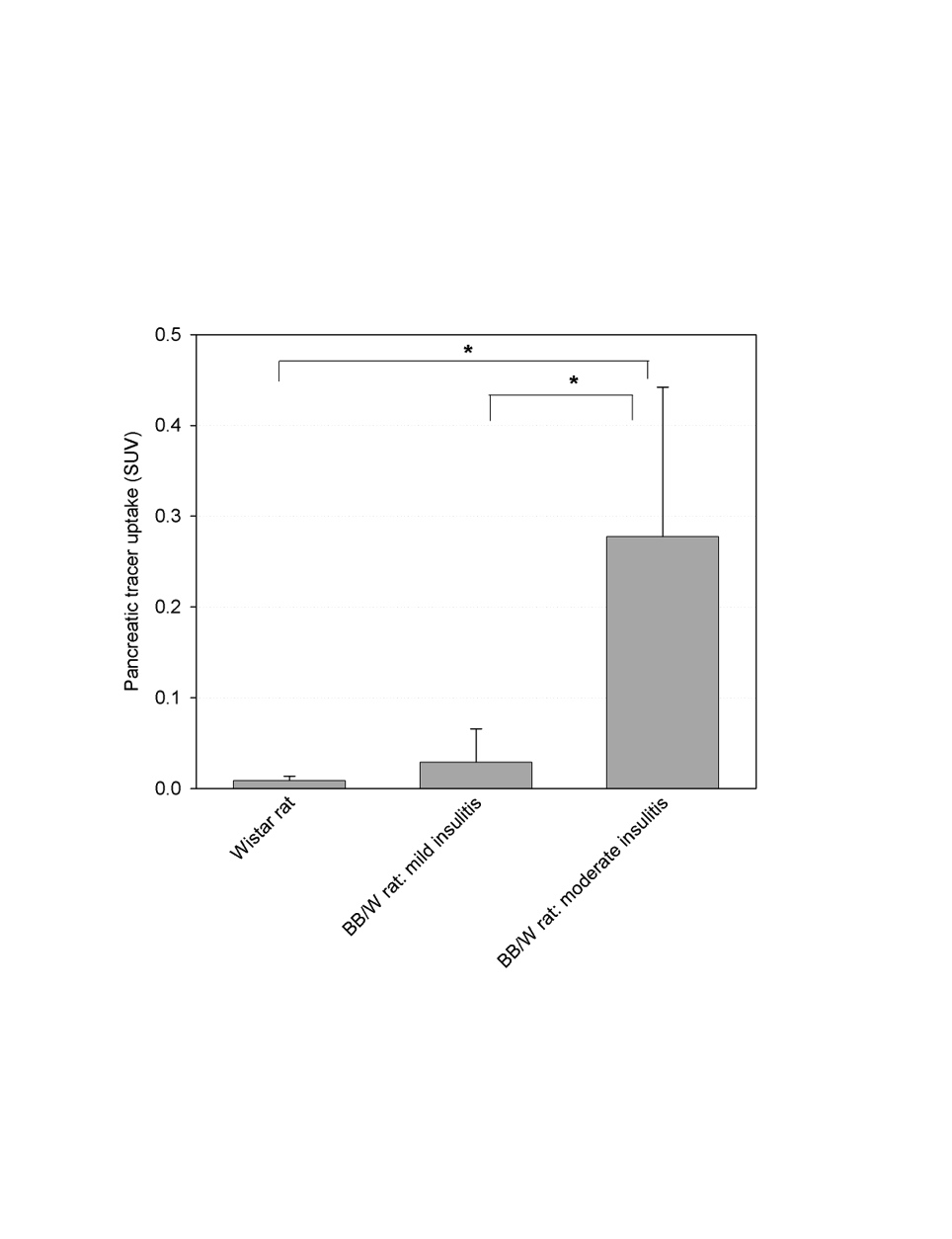


*Figure 10:Coronal (A) and transaxial (B) [18F]FB-IL2 PET images of a diabetic-prone BB/W rat, showing high uptake in the infiltrated pancreas (left). Tracer uptake was highly dependent on the extent of insulitis (i.e. the amount of infiltrating T lymphocytes) (right)*

Tumor-infiltrating T lymphocytes

Tumor infiltration in response to radiotherapy and/or immunization was previously evaluated in tumor-bearing mice using flow cytometry [3]. We used the same experimental design to investigate the feasibility of [^18^F]FB-IL2 PET imaging to monitor the effect of radiotherapy, alone or in combination with immunization, on the tumor infiltration of activated T-lymphocytes [8]. In addition, we investigated whether the infiltration of lymphocytes is mediated by the CXCR4 receptor signaling pathway. For this purpose, we used [^18^F]FB-IL2 PET to monitor the tumor infiltration of T lymphocytes in HPV-positive TC-1 tumors in mice. Tumor irradiation with a single 14 Gy dose enhances the influx of activated T cells into the tumor. PET imaging studies showed that [^18^F]FB-IL2 uptake was significantly increased in irradiated tumors (3.34±0.7 %ID/g), when compared to controls (0.34±0.16 %ID/g, p<0.01). Combination of tumor irradiation with immunization even further increases tumor infiltration (9.21±1.82.7 %ID/g, p<0.001). Inhibition of the CXCR4 signaling pathway with the antagonist AMD3100 significantly reduced the increased [^18^F]FB-IL2 uptake in irradiated animals (1.32±0.26 %ID/g, p<0.01). Interestingly, ex-vivo biodistribution studies showed that immunization – but not irradiation alone – also significantly increased the [^18^F]FB-IL2 uptake in immune-related organs, such spleen (5-fold, p<0.001), salivary gland (5-fold, p<0.001), lymph nodes (6-fold, p<0.001) and bone marrow (7-fold, p<0.05). In addition, combined irradiation and immunization resulted in a significantly increased tracer uptake in lungs, thymus (p<0.01), blood, plasma and liver (p<0.05), when compared to irradiation alone. Taken together, these data demonstrate that [^18^F]FB-IL2 PET can detect changes in T cell activation and infiltration in the tumor in response to treatment. Moreover, [^18^F]FB-IL2 PET also detected T cell activation in other tissues than the tumor, suggesting that the technique may also be used to detect potential adverse events before they become overt.

### 2.2.2 Non-clinical pharmacokinetics

Plasma kinetics

The pharmacokinetic profile of proleukin® following i.v. administration in Wistar rats is characterized by a high plasma concentration that declines in a bi-modal exponential form. The initial elimination of IL-2, which is mainly due to extracellular distribution and renal elimination, has been reported to have a plasma half-life (T_1/2_) of 7-14 minutes. The second phase, attributed solely to excretion via the kidneys is slower and estimated to have a terminal T_1/2_ of 85 minutes (3). Similarly, ^99m^Tc-labeled IL-2 biodistribution and pharmacokinetic studies in humans have shown a rapid and bi-modal plasma clearance with T_1/2_ = 5.7±0.2 min and T_1/2_ = 121±6 min for first and second phase, respectively. Furthermore, biodistribution and kinetics of ^99m^Tc-labeled IL-2 were found to be similar to those of ^123^I-labeled IL-2 (4). The blood clearance of [^18^F]FB-IL2 in Wistar rats with a xenograft of human PBMCs showed a two-phase blood clearance with a half-life of 0.7±0.3 minutes and 8.4±2.6 minutes for the first and second phase, respectively (Figure 11), which corresponds well with the first phase observed for native IL2 [6].

Metabolism

Metabolite analysis in plasma samples from Wistar rats showed that [^18^F]FB-IL2 is highly stable in plasma, as the fraction of intact [^18^F]FB-IL2 in plasma was still 97.7±1.2% at 60 minutes after tracer injection [6]. In contrast, metabolite analysis of urine samples demonstrated that the main excretion product is an unknown hydrophilic degradation product of [^18^F]FB-IL2. The amount of intact [^18^F]FB-IL2 in urine is negligible. These results indicate that [^18^F]FB-IL2 is degraded in the kidneys before it is excreted into the urine, which is similar to native IL2.

Pharmacokinetic profile

Pharmacokinetic modeling studies were performed in Wistar rats with PHA-activated human PBMC xenografts [6]. Tracer uptake in the PBMC xenograft displayed a one-phase clearance curve with a half-life of 37±4 min. The pharmacokinetic profile of [^18^F]FB-IL2 in the xenograft was well described by Logan graphical analysis, but not by Patlak analysis, indicating that tracer binding is reversible. Pharmacokinetic modeling showed that tracer uptake in the xenograft could be best described by a reversible two-tissue compartment model. This model was used to calculate the binding potential (B_max_/K_D_ = k_on_/k_off_) of [^18^F]FB-IL2. There is an excellent correlation between the binding potential of [^18^F]FB-IL2 and the number of IL2 receptor-positive (CD25+) cells in the xenograft (r^2^ 0.88, p<0.0001). The binding potential increases by a factor of 4 for each million cells present in the inflammatory lesion.


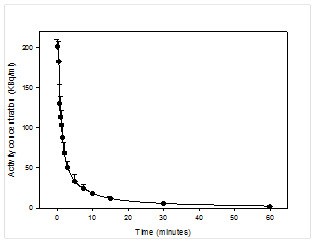


*Figure 11:Time-activity curves of [18F]FB-IL2 in plasma (left) and a PBMC xenograft (right) in Wistar rats.*

Limit of Detection

The limit of detection of [^18^F]FB-IL2 PET was determined in Wistar rats with human PBMC xenografts by comparing the binding potential from a control area (i.e. contra-lateral shoulder) with that of the inoculation site [6]. In this animal model, the lowest number of CD25-positive cells that can be reliably (95% confidence) detected by this methods, is approximately 160,000 cells in a lesion with a volume of 200 µL. The data also suggested that [^18^F]FB-IL2 PET is a potentially more sensitive tool to measure activated T lymphocytes than radiolabeled IL-2 analogues that were previously described for SPECT imaging.

### 2.2.3 Test Materials used in toxicity studies

Not applicable.

### 2.2.4 Integrated assessment of the data package

[^18^F]FB-IL2 showed favorable binding parameters to the IL2 receptor and in-vivo kinetics. The tracer is stable in plasma, rapidly cleared from non-target tissues and excreted into the urine. Because of the low background signal, high contrast images of activated T-cells can be obtained. The feasibility of imaging of T-cell infiltration with [^18^F]FB-IL2 PET has been clearly demonstrated in animal models for autoimmune diseases and in tumor-bearing mice treated with radiotherapy and/or immunization.

In animal studies, [^18^F]FB-IL2 shows similar behavior, kinetic profile and binding characteristics as Proleukin®. Non-clinical toxicity studies have not been performed for [^18^F]FB-IL2. However, the predictive value of such toxicity tests would be highly questionable, because a human protein is used in a rodent species. The test may be false positive due to immune response, or false negative due to species differences in receptor affinity. The toxicity profile of IL2 in humans and animals is well known and it is reasonable to assume that the toxicity profile of [^18^F]FB-IL2 is similar to Proleukin®, especially since the perturbation of the protein structure is minimal (label 122D vs. protein 15,300D). [^18^F]FB-IL2 will be administered as a single sub-pharmacological dose equal to or less than 50 µg, and is therefore unlikely to pose a toxicological threat in micro-dose [^18^F]FB-IL2 PET imaging studies. Because of the fast clearance of [^18^F]FB-IL2, the residence time of the tracer in the body will be short.

### 2.2.5 List of studies Conducted & References

*Table 7:List of non-clinical studies for [^18^F]BF-IL2*

| **Study or Report Number** | **Author(s)** | **Title of report** |
| --- | --- | --- |
| Ref. (5) | Di Gialleonardo et al. | N-(4-^18^F-fluorobenzoyl)interleukin-2 for PET of human-activated T lymphocytes. |
| Ref. (6) | Di Gialleonardo et al. | Pharmacokinetic modelling of N-(4-[(^18^)F]fluorobenzoyl)interleukin-2 binding to activated lymphocytes in an xenograft model of inflammation. |
| Ref. (7) | Di Gialleonardo et al. | N-(4-[^18^F]fluorobenzyl)interleukin-2, a new PET radiopharmaceutical for imaging of insulitis |
| Ref. (8) | Hartimath et al. | Monitoring the effect of tumor irradiation and immunization on CXCR4 expression and tumor infiltrating lymphocytes with PET |

### 2.2.6 GLP Statement and bioanalytical methods

Not applicable.

## 2.3 CLINICAL DATA

### 2.3.1 Clinical pharmacology

Clinical data for IL2 are described in appendix A, summary of product characteristics.

Several clinical imaging studies, in which IL2 radiolabeled with ^99m^Tc or ^123^I was used, have been described [1-4, 9-17]. ^99m^Tc-labeled HYNIC-IL2 has already been used in clinical studies within the UMCG. In these studies, ^99m^Tc-labeled HYNIC-IL2 was administered in a sub-pharmacological dose. Consequently, no adverse effects from this dose of ^99m^Tc-HYNIC-IL2 were seen in previous studies. A little decrease in peripheral blood lymphocytes was observed in the first hours after administration of the radiopharmaceutical in patients with autoimmune diseases, but not in healthy volunteers. However, these hematological changes were mild and transient. Given the fact that the pharmacology of the tracer will be predominantly determined by the properties of the protein, rather than the relatively small label that is attached to it, it can be expected that the label will not substantially affect the properties of the tracers and thus that the pharmacology of all radiolabeled IL2 derivatives will be comparable and similar to naïve IL2.

Clinical pharmacology data for [^18^F]FB-IL2 are scarce yet, since the first clinical trials, in which this PET tracer is used, are still on-going (26-6-2018). So far, 18 [^18^F]FB-IL2 PET scans have been performed and no adverse events related to the use of the PET tracer have been observed in these trials.

### 2.3.2. Clinical pharmacokinetics

Clinical data for IL2 are described in appendix B, brochure (IB-1) of Proleukin®.

Data from imaging studies with IL2 radiolabeled with ^99m^Tc or ^123^I are available [1-4, 9-17]. The most relevant pharmacokinetic data of the radiolabeled conjugate ^99m^Tc-HYNIC-IL2 are briefly discussed below. Studies in normal subjects showed fast plasma clearance of ^99m^Tc-IL2 (Table 8). Kidneys were the major organs of accumulation of ^99m^Tc-IL2; the uptake increased up to 1 hour and declined thereafter. Liver and spleen also exhibited detectable uptake of the radiopharmaceutical. Some excretion in the bowel, but no uptake in the thyroid or any other organ was observed (thyroid to background ratio: 1.06±0.05) [10]. Liver and bowel uptake is likely due to free ^99m^Tc that is released from the protein complex, rather than radiolabeled protein. Trichloroacetic acid (TCA) precipitation of plasma showed that most circulating radioactivity at 1 and 4 hour was associated with ^99m^Tc-IL2. TCA precipitation of the urine showed a lower degree of protein-bound radioactivity compared to plasma. Cumulative urinary excretion of ^99m^Tc-IL2 four hours p.i. was 26±5.7% of administered activity [10]. Analysis of the distribution of radioactivity within whole blood revealed that 1.3% was associated with the white blood cells, 10% with the red blood cells and 88.7% with the plasma [10].

Studies in human healthy volunteers, showed high in vivo stability of ^99m^Tc-IL2, higher than previously shown with ^123^I-IL2. In addition, rapid plasma clearance and low background radioactivity, comparable to that of ^123^I-IL2, were observed. Kidneys were the major organs of accumulation of ^99m^Tc-IL2, which was excreted in the urine mostly in the form of low molecular weight metabolites confirming previous studies in animals that demonstrated that IL2 is mainly metabolized in the kidneys. No intestinal excretion was observed.

Compared to normal subjects, studies in patients with autoimmune diseases displayed an increased level of accumulation of ^99m^Tc-IL2 in the liver and, to a lesser extent, in the spleen [10]. More than one mechanism could be advocated for this phenomenon. It is possible that, following the state of immune activation in patients with autoimmune diseases, a greater number of circulating IL2 receptor-positive cells or a higher level of expression of the IL2 receptor is observed in lymphocytes homing into the liver and in the spleen. The binding of ^99m^Tc-IL2 to the soluble IL2 receptor, that has previously accumulated in the liver, is also possible, as well as liver uptake of circulating ^99m^Tc-IL2 / soluble IL2 receptor complexes. The sensitivity of ^99m^Tc-IL2 in pathologies of the liver and kidneys may, therefore, be reduced owing to its accumulation in the absence of local pathology.

*Table 8:Rate of clearance of 99mTc-IL2 from different organs and tissues (minutes) [10].*

|  | **alpha phase** | **beta phase** |
| --- | --- | --- |
| plasma | 5.7±0.2 | 121±5.8 |
| kidneys |  | 186±31.2 |
| liver |  | 119.4±19.8 |
| spleen |  | 115.2±36 |

Clinical pharmacokinetics data for [^18^F]FB-IL2 are scarce yet, since the first clinical trials, in which this PET tracer is used, are still on-going. In melanoma patients highest uptake was observed in liver, spleen, kidney and bladder content at 60 and 120 post tracer injection. Intermediate uptake was observed in bone marrow, whereas other tissues showed very low uptake. Clearance rates of [^18^F]FB-IL2 from blood appear to be slightly faster than for ^99m^Tc-IL2, with halflives of approximately 3 min and 1 hour for the alpha and beta phase, respectively. Hardly any metabolites of [^18^F]FB-IL2 were observed in blood, with >90% of radioactivity in plasma still consisting of intact tracer at 60 min after injection.

### 2.3.4 Human exposure

Risks and side-effects of a pharmacological dose of IL-2 are described in appendix A. [^18^F]FB-IL2 will be administered in a sub-pharmacological dose (≤50 µg versus 1,1 mg used per clinical dose). Therefore adverse effects are not to be expected. In addition, no toxic effects were observed in previous studies with radiolabeled IL2. Over 600 patients have been studied in the setting of inflammatory bowel diseases, autoimmune thyroid diseases, insulin dependent diabetes, melanoma, atherosclerotic plaques and other different pathologies. Hematological changes were within normal limits and transient.

However, the use of radioactive isotopes means exposure to ionizing radiation. Because of the potential hazards of radiation, guidelines for the exposure of healthy volunteers are specified in “Besluit Stralingsbescherming (BS 2000), artikel 60, Staatsblad 2001, 397", according to the guidelines of the International Commission on Radiological Protection (ICRP 80).

The effective dose equivalent of [^18^F]FB-IL2 in humans has not been measured yet. The effective dose equivalent of ^99m^Tc-IL2 was calculated to be 7.3 μSv/MBq, i.e. 1.35 mSv for a typical diagnostic scan (185 MBq) [10]. The effective dose equivalent of [^18^F]FB-IL2 can be estimated based on available data for other [^18^F] tracers, or with the assumption that [^18^F]FB-IL2 has a homogenous distribution throughout the body. The effective dose equivalent of [^18^F] tracers was estimated to be ca. 0.02 mSv/MBq. For a diagnostic dose of [^18^F]FB-IL2 equal to 200 MBq, the absorbed radiation dose will be ca. 4 mSv. Thus, the radiation burden of a single administration of 200 MBq [^18^F]FB-IL2 can be classified as a category IIB risk (low to intermediate), as described in communication 62 by the International Committee on Radiological Protection (ICRP62).

## 2.4 Overall risk and benefit assessment

[^18^F]FB-IL2 binds to high-affinity IL2 receptors expressed on activated T-lymphocytes and monocytes in tumors and inflamed sites and could be used to monitor tumor infiltration of activated T-cells. In this way, [^18^F]FB-IL2 PET can provide information about the drug-induced activation of immune cells within all tumor lesions within the patient and thus could be used as an early marker for treatment response. Moreover, [^18^F]FB-IL2 PET could also detect infiltration of activated T cells in normal organs, which might potentially give rise to immune-related adverse effects. This information can currently not be obtained in any other manner. Since [^18^F]FB-IL2 is administered in a sub-pharmacological dose, the pharmacological effects are expected to be minimal. This was confirmed in previous clinical studies with IL2 radiolabeled with ^123^I or ^99m^Tc in patients, in which no adverse effects of the tracer were observed. Labeling of IL2 with ^123^I or ^99m^Tc causes larger perturbation of the protein structure than labeling with ^18^F as in [^18^F]FB-IL2.

[^18^F]FB-IL2 has the tendency to adhere to disposable materials like filters, lines and syringes and to form reversible aggregated. To avoid this, excipients have been added to the formulation, but as a result the osmolarity of final formulation is relatively high. To reduce the impact of the osmolarity the volume of the injected tracer solution will be restricted to approximately 10 mL and the solution will be injected slowly over a period of 5 min.

The radiation burden of a diagnostic dose of [^18^F]FB-IL2 is estimated as low to moderate, which is within the normal ranges of nuclear medicine applications. In the first patients that have been investigated with [^18^F]FB-IL2, no adverse effects were observed. Therefore, it can be expected that the use of [^18^F]FB-IL2 is safe and potentially useful for the study of inflammatory processes in various diseases.

References

1. Signore A, Annovazzi A, Bonanno E, D’Alessandria C, Chianelli M, Mather S, Bottoni U, Panetta C, Innocenzi D, Scopinaro F, Calvieri S. 99mTc-Interlukin-2 scintigraphy as a potential tool for evaluating tumor-infiltrating lymphocytes in melanoma lesions: a validation study. J Nucl Med 2004 Oct:45:1647-1652.
2. Loose D, Signore A, Staelens L, Bulcke KV, Vermeersch H, Dierckx RA, Bonanno E, Van de Wiele C. (123)I-Interleukin-2 uptake in squamous cell carcinoma of the head and neck carcinoma. *Eur J Nucl Med Mol Imaging.* 2008; 35(2):281-6.
3. Signore A, Picarelli A, Annovazzi A, Britton KE, Grossman AB, Bonanno E, Maras B, Barra D, Pozzilli P. ^123^I-interlukin-2: biochemical characterization and in vivo use for imaging autoimmune diseases. *Nuc Med Commun* 24: 305-316; 2003
4. D'Alessandria C, di Gialleonardo V, Chianelli M, Mather SJ, de Vries EF, Scopinaro F, Dierck RA, Signore A. Synthesis and optimization of the labeling procedure of 99mTc-HYNIC-interleukin-2 for in vivo imaging of activated T lymphocytes. Mol Imaging Biol. 2010 Oct;12(5):539-46.
5. Di Gialleonardo V, Signore A, Glaudemans AW, Dierckx RA, De Vries EF. N-(4-18F-fluorobenzoyl)interleukin-2 for PET of human-activated T lymphocytes. J Nucl Med. 2012 May;53(5):679-86.
6. Di Gialleonardo V, Signore A, Willemsen AT, Sijbesma JW, Dierckx RA, de Vries EF. Pharmacokinetic modelling of N-(4-[(18)F]fluorobenzoyl)interleukin-2 binding to activated lymphocytes in an xenograft model of inflammation. Eur J Nucl Med Mol Imaging. 2012 Oct;39(10):1551-60.
7. Di Gialleonardo V, de Vries EFJ, Glaudemans AWJM, Chianelli M, Wolffenbuttel BHR, de VosP, Visser JTJ, Dierckx RAJO, Signore A. Evaluation of Nlys-(4-[18F]fluorobenzoyl)-interleukin-2 as a diagnostic probe for PET imaging of insulitis in NOD mice and BB/W rats. Submitted for publication.
8. Hartimath SV, Draghiciu O, Manuelli V, Dierckx RAJO, Nijman HW, Daemen T, de Vries EFJ. Monitoring the effect of tumor irradiation and immunization on CXCR4 expression and tumor infiltrating lymphocytes with PET. Oncoimmunology. 2016 Nov 18;6(1):e1248014.
9. Shaker MA, Younes HM. Interleukin-2: evaluation of routes of administration and current delivery systems in cancer therapy. J Pharm Sci. 2009 Jul;98(7):2268-98.
10. M. Chianelli, S.J. Mather, A. Grossman, R. Sobnak, A. Fritzberg, K.E. Britton, A. Signore. ^99m^Tc-Interleukin-2 scintigraphy in normal subjects and in patients with autoimmune thyroid diseaseS: a feasibility study. Eur J Nucl Med Mol Imaging. 2008 Dec;35(12):2286-93.
11. Chianelli M, Parisella MG, Visalli N, Mather SJ, D'Alessandria C, Pozzilli P, Signore A; IMDIAB study group. Pancreatic scintigraphy with 99mTc-interleukin-2 at diagnosis of type 1 diabetes and after 1 year of nicotinamide therapy. Diabetes Metab Res Rev. 2008; 24(2):115-22.
12. Renard V, Staelens L, Signore A, Van Belle S, Dierckx RA, Van De Wiele C. Iodine-123-interleukin-2 scintigraphy in metastatic hypernephroma: a pilot study. Q J Nucl Med Mol Imaging. 2007;51(4):352-6.
13. Annovazzi A, Bonanno E, Arca M, D’Alessandria C, Marcoccia A, Spagnoli LG, Violi F, Scopinaro F, De Toma G, Signore A. ^99m^Tc-interleukin-2 scintigraphy for the in vivo imaging of vulnerable atherosclerotic plaques. Eur J Nucl Med Mol Imaging 2006. 33:117-126.
14. Lucia P, Parisella MG, Danese C, Bruno F, Manetti LL, Capriotti G, De Martinis C, Scopinaro F, Perego MA, Signore A. Diagnosis and followup of Takayasu's arteritis by scintigraphy with radiolabelled interleukin 2. J Rheumatol. 2004 Jun;31(6):1225-7.
15. Annovazzi A, Biancone L, Caviglia R, Chianelli M, Capriotti G, Mather SJ, Caprilli R, Pallone F, Scopinaro F, Signore A. ^99m^Tc-interleukin-2 and ^99m^Tc-HMPAO granulocyte scintigraphy in patients with inactive Crohn's disease. Eur J Nucl Med Mol Imaging 30: 374-382; 2003.
16. Signore A, Chianelli M, Annovazzi A, Bonanno E, Spagnoli L, Pozzilli P, Pallone F, Biancone L. ^123^I-Interleukin-2 scintigraphy for the in vivo assessment of intestinal mononuclear cell infiltration in Chron's disease. J Nucl Med 41:242-249;2000.
17. Signore A, Chianelli M, Annovazzi A, Rossi M, Maiuri L, Greco M, Ronga G, Britton KE, Picarelli A. Imaging of active lymphocytic infiltration in coeliac disease with ^123^I-Interleukin-2 and its response to diet. Eur J Nucl Med 27:18-24;2000.

**Appendix**

Appendix A: Summary of product specifications Proleukin® (SmPC Proleukin® “Samenvatting van productkenmerken”).
